# Distinct cardiac energy metabolism and oxidative stress adaptations between obese and non-obese type 2 diabetes mellitus

**DOI:** 10.7150/thno.40735

**Published:** 2020-02-03

**Authors:** Xinghui Li, Yandi Wu, Jingjing Zhao, Haiping Wang, Jing Tan, Ming Yang, Yuanlong Li, Shijie Deng, Saifei Gao, Hui Li, Zhenyu Yang, Fengmin Yang, Jianxing Ma, Jianding Cheng, Weibin Cai

**Affiliations:** 1 Guangdong Engineering & Technology Research Center for Disease-Model Animals, Laboratory Animal Center, Zhongshan School of Medicine, Sun Yat-sen University, Guangzhou 510080, Guangdong, China.; 2 Department of Biochemistry, Zhongshan School of Medicine, Sun Yat-sen University, Guangzhou 510080, Guangdong, China.; 3 NHC Key Laboratory on Assisted Circulation, Department of Cardiology, the First Affiliated Hospital, Sun Yat-Sen University, Guangzhou 510080, Guangdong, China.; 4 Department of Physiology, Health Sciences Center, University of Oklahoma, Oklahoma City, OK 73104, USA.; 5 Department of Forensic Pathology, Zhongshan School of Medicine, Sun Yat-sen University, Guangzhou 510080, Guangdong, China.

## Abstract

**Background**: Little is known about the pathophysiological diversity of myocardial injury in type 2 diabetes mellitus (T2DM), but analyzing these differences is important for the accurate diagnosis and precise treatment of diabetic cardiomyopathy. This study aimed to elucidate the key cardiac pathophysiological differences in myocardial injury between obese and non-obese T2DM from mice to humans.

**Methods**: Obese and non-obese T2DM mouse models were successfully constructed and observed until systolic dysfunction occurred. Changes in cardiac structure, function, energy metabolism and oxidative stress were assessed by biochemical and pathological tests, echocardiography, free fatty acids (FFAs) uptake fluorescence imaging, transmission electron microscopy, *etc*. Key molecule changes were screened and verified by RNA sequencing, quantitative real-time polymerase chain reaction and western blotting. Further, 28 human heart samples of healthy population and T2DM patients were collected to observe the cardiac remodeling, energy metabolism and oxidative stress adaptations as measured by pathological and immunohistochemistry tests.

**Results**: Obese T2DM mice exhibited more severe cardiac structure remodeling and earlier systolic dysfunction than non-obese mice. Moreover, obese T2DM mice exhibited severe and persistent myocardial lipotoxicity, mainly manifested by increased FFAs uptake, accumulation of lipid droplets and glycogen, accompanied by continuous activation of the peroxisome proliferator activated receptor alpha (PPARα) pathway and phosphorylated glycogen synthase kinase 3 beta (p-GSK-3β), and sustained inhibition of glucose transport protein 4 (GLUT4) and adipose triglyceride lipase (ATGL), whereas non-obese mice showed no myocardial lipotoxicity characteristics at systolic dysfunction stage, accompanied by the restored PPARα pathway and GLUT4, sustained inhibition of p-GSK-3β and activation of ATGL. Additionally, both obese and non-obese T2DM mice showed significant accumulation of reactive oxygen species (ROS) when systolic dysfunction occurred, but the NF-E2-related factor 2 (Nrf2) pathway was significantly activated in obese mice, while was significantly inhibited in non-obese mice. Furthermore, the key differences found in animals were reliably verified in human samples.

**Conclusion**: Myocardial injury in obese and non-obese T2DM may represent two different types of complications. Obese T2DM individuals, compared to non-obese individuals, are more prone to develop cardiac systolic dysfunction due to severe and persistent myocardial lipotoxicity. Additionally, anti-oxidative dysfunction may be a key factor leading to myocardial injury in non-obese T2DM.

## Introduction

With the increasing number of clinical treatment strategies for diabetes in recent years, the quality of life and life expectancy of diabetic patients have improved significantly 1. However, in this process, the cardiovascular complications of diabetes have been highlighted due to their occult nature and high mortality. Currently, 50-80% of diabetic patients eventually die of cardiovascular disease, and the risk of heart failure in diabetic patients is 2-4-fold higher than that of nondiabetic individuals 1-3. Diabetic cardiomyopathy (DCM), a serious cardiovascular complication of diabetes characterized by cardiac structure remodeling and dysfunction, is related to various pathological mechanisms involving myocardial lipotoxicity, glucose toxicity, oxidative stress, apoptosis, autophagy, *etc* 4-7. However, strategies to prevent or ameliorate myocardial injury in diabetic patients are still scarce.

Obviously, an analysis of the pathophysiological differences in diabetic myocardial injury is the basis for molecular typing, accurate diagnosis and precise treatment of DCM, which would reduce cardiovascular events in people with diabetes. Additionally, an increasing number of studies has suggested that diabetes and its complications have multiple complex manifestations; therefore, more precise classification is needed to ensure that patients receive appropriate treatment 8-10. Obesity, as an independent risk factor for cardiovascular disease and an important factor leading to the high incidence of type 2 diabetes mellitus (T2DM), is also a major contributor to DCM 11-14. However, a portion of patients with DCM have normal or below-normal body weight. Additionally, significant differences exist in the therapeutic effects of the clinical treatment of DCM between obese and non-obese T2DM patients, although the underlying causes of this difference are unknown; differences in body mass index (BMI) may be a key contributor to these differences 15. Previous clinical studies have shown that obese T2DM patients exhibit more severe ectopic and visceral fat deposition than non-obese T2DM patients, and this condition is associated with cardiac contractile dysfunction 16. Thus, we speculate that obese and non-obese T2DM patients with DCM may exhibit pathophysiological differences, and therefore, these groups of patients should be treated with different strategies. Although increasing research has focused on the differences in DCM between type 1 and 2 diabetes mellitus, research on the differences in DCM between obese and non-obese T2DM patients is rare 17-20.

**Figure 1:**
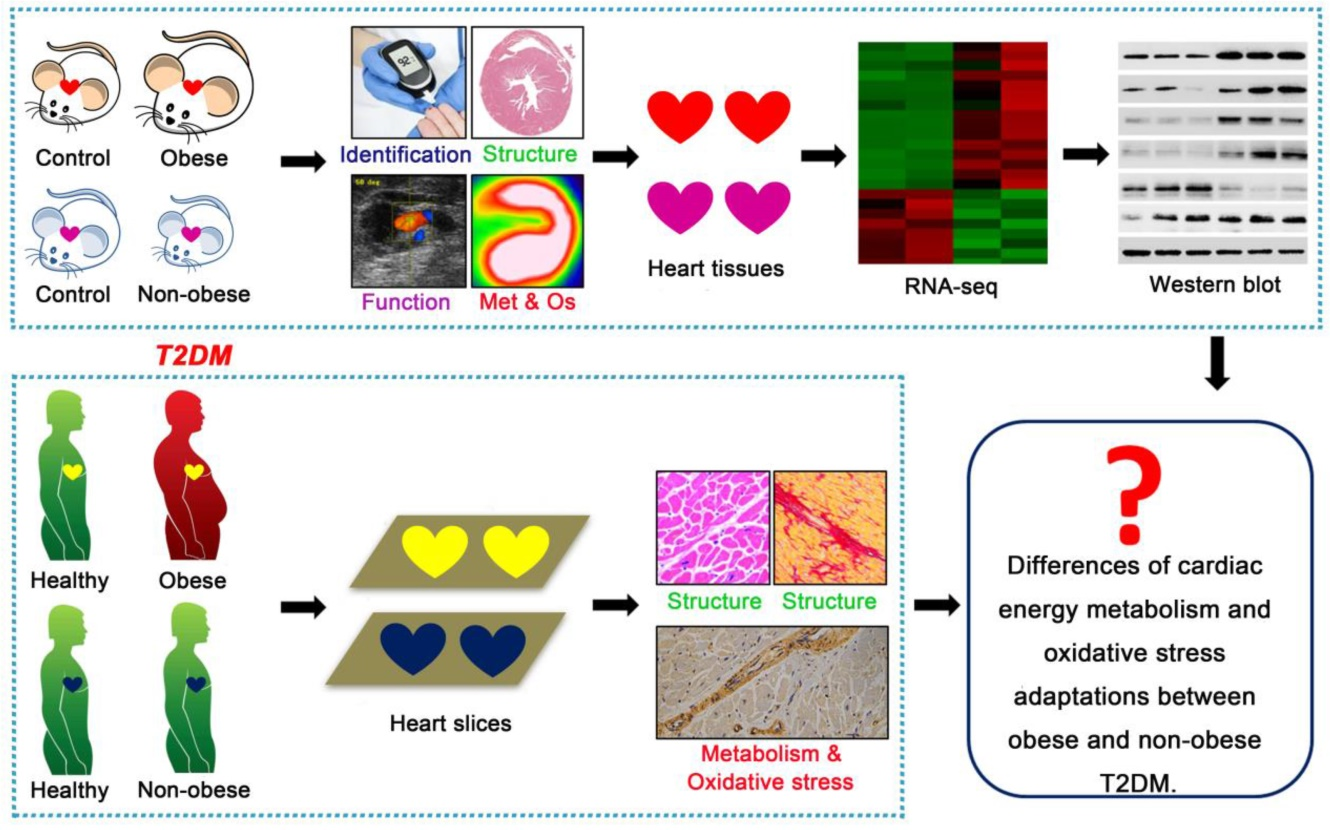
** Technical roadmap of the study.** Obese and non-obese T2DM mouse models were successfully constructed and the cardiac structure, function, metabolism (Met), oxidative stress (Os) and related molecular changes were systematically observed. Human heart samples of healthy population and T2DM patients were collected to observe the cardiac remodeling, energy metabolism and oxidative stress adaptations.

This study aimed to elucidate the key cardiac pathophysiological differences in myocardial injury between obese and non-obese T2DM patients from mice to humans. *Db/db* mice is a classic genetically modified mouse model that is widely used as an animal model for studying T2DM; this model features obesity, insulin resistance, hyperglycemia, hyperlipidemia and manifestations of cardiomyopathy 21. Streptozotocin (STZ) is an antibiotic that can selectively destroy pancreatic islet β-cells 22. As a common method for the chemical induction of diabetes mellitus, one single high-dose STZ injection into mice or rats can completely destroy islet β-cells, resulting in a type 1 diabetes phenotype; conversely, multiple low-dose STZ injections with high fat diet (HFD) can induce insulin resistance, resulting in a T2DM phenotype 22. Changes in the body weights of animals treated with STZ depend on the injection dose, injection time and drug resistance of different animal strains 23,24. In this study, we used *db/db* mice as an animal model for T2DM with obesity. A low-dose STZ injection combined with a HFD was used to induce the animal model of non-obese T2DM. Then, we systematically observed the changes in cardiac structure and function, energy metabolism, oxidative stress and molecular mechanisms during the course of diabetes mellitus in both models, and we analyzed the phenotypic and mechanistic differences in DCM between the models. Further, we validated the relevant mechanisms (cardiac energy metabolism and oxidative stress adaptations) and clarified our conclusions using human samples, including those collected from obese and non-obese T2DM patients and from healthy individuals (Figure 1). We expected this study to reveal the key cardiac pathophysiological differences between obese and non-obese T2DM patients. Importantly, an understanding of the pivotal pathophysiological differences between obese and non-obese T2DM patients will provide a theoretical and molecular basis for the accurate diagnosis and precise treatment of DCM in T2DM patients and enable the development of more targeted treatments for T2DM patients based on BMI to ameliorate myocardial injury.

## Methods

### Animal model and treatment

*Db/m* (C57BLKS/J background) mice were purchased from The Jackson Laboratory following breeding and expansion of a population from the Center for Disease Model Animals of Sun Yat-sen University. Eight-weeks-old male *db/db* mice and *db/m* mice (30 animals per group) were used in this study to build the model of T2DM with obese. Eight-weeks-old *db/db* mice were defined as the baseline for the duration of diabetes mellitus, and the duration of our observation lasted until the 24th week of the disease course. All animals were given water and chow diet during the whole experiment period. During this period, body weight, fasting blood glucose (FBG) and echocardiography were measured every 4 weeks. At 4, 12, 20 and 24 weeks of the disease course, blood and heart tissues were collected and serum was separated, serum insulin content, serum lipid content, heart weight, tibia length were measured. The insulin tolerance test (ITT) was performed at 4 weeks of disease course, tissues free fatty acids (FFAs) uptake fluorescence imaging were performed at 24 weeks of disease course. Some heart tissues were fixed and embedded for subsequent detection. All serum and tissue samples were stored at -80 °C.

Non-obese T2DM mouse model were constructed using a method described previously with minor modifications 25-27. Four-weeks-old male C57BL/6J mice were purchased from Laboratory Animal Center of Sun Yat-sen University. Eighty animals (30 animals were used as the control group and 50 animals were used to induce T2DM as the model group) were used in this study. To induce diabetes, mice of the model group were treated with HFD at 4-weeks-old and were treated with seven consecutive intravenous injections of STZ (40 mg/kg, Sigma, St. Louis, MO) in citrate buffer (pH 4.6) at 8-weeks-old, while the control animals received chow diet and the same volume of citrate buffer. The animals of model group were given 10% sucrose/water during the period from 12 h after the first STZ injection to 12 h after the last injection and were given HFD during the whole experiment period. The animals of control group were given water and chow diet during the whole experiment period. The blood glucose level was monitored with a glucometer (One Touch Ultra Easy, Life Scan, PA, USA), on 2 weeks after the last STZ injection, and animals with blood glucose levels greater than 12 mmol/L were considered diabetic. This time point was defined as the baseline for the duration of diabetes mellitus, and the duration of our observation lasted until the 36th week of the disease course. During this period, body weight, FBG and echocardiography were measured every 4 weeks. At 4, 12, 24 and 36 weeks of the disease course, blood and heart tissues were collected and serum was separated, serum insulin content, serum lipid content, heart weight, tibia length were measured. The ITT and glucose tolerance test (GTT) were performed at 4 weeks or 24 weeks of the disease course, tissues FFAs uptake fluorescence imaging were performed at 36 weeks of the disease course. Some heart tissues were fixed and embedded for subsequent detection. All serum and tissue samples were stored at -80 °C.

All animals were kept under 12-h light-dark cycles at 20-22 °C with free access to water and food. All animals were fasted for 12-16 h before sampling and testing. All studies involving animal experimentation were approved by the Animal Care and Ethics Committee of Sun Yat-sen University and followed the National Institutes of Health Guidelines on the Care and Use of Animals.

### Echocardiography

Transthoracic echocardiography was performed on mice at the indicated time points using a Vevo 2100 imaging system (Visual Sonics, Toronto, Canada). In detail, the mice to be subjected to echocardiography were placed in a closed box filled with 1.5% isoflurane. After being anesthetized, the mice were immediately placed on a 37 °C thermostat to maintain normal body temperature and maintained on 0.5% isoflurane to prevent them from waking up until the end of the imaging process. For the image acquisition process, a 30-MHz probe was used first to detect the heart motion in the long-axis view. Then, the probe was rotated 90 degrees to detect the heart motion in the short-axis view, and the rheography was captured in M-Mode at the papillary muscle level. Then, the probe position was changed, and the mode was switched to Color-Mode to detect the mitral valve blood flow rheography, and the rheography was captured in PW-Mode. The imaging times for M-Mode and Doppler-Mode were approximately 1 min and 5 min, respectively. In addition, during the imaging process, the heart rate of the mouse was continuously detected and maintained in a stable state. Except for non-obese diabetic mice with a course of 36 weeks, the HR of other mice was controlled at 450-550 bpm during imaging, while for 36-week course of non-obese diabetic mice, its HR is controlled at 200-250 bpm during imaging because their HR was only 410-430 bpm in the awake state (as detected by noninvasive blood pressure measurement system (BP-98A, Softron, Tokyo, Japan)). Such heart rate control can ensure that the assessment of cardiac function in mice is close to that in the awake state.

### Serum measurement

Serum were obtained by centrifugation of clotted blood collected from the eye sockets of the mice and stored at -80 °C. The serum fasting insulin (FINS) levels were examined using an enzyme-linked immunosorbent assay (Cusabio, Wuhan, Hubei, China). The serum FFAs, triglycerides (TG), total cholesterol (TC), low-density lipoprotein (LDL) and high-density lipoprotein (HDL) levels were examined using commercial reagent kits (Jiancheng, Nanjing, Jiangsu, China).

### Homeostasis model assessment of the insulin resistance index (HOMA-IR), ITT and GTT

The HOMA-IR was calculated with the equation [FBG(mmol/L)*FINS(mIU/L)]/22.5. To perform the ITT and GTT, 1 U/kg of insulin (Novolin R, Novo Nordisk, Bagsvaerd, Denmark) or 1 g/kg of glucose (Sigma-Aldrich, St. Louis, MO, USA) was i.p. injected into the mice had fasted for 12-16 h. The blood glucose levels were examined 0, 15, 30, 60 and 120 min after the injection. The curve of the blood glucose change level was drawn, and the area under the curve (AUC) was calculated.

### Tissues FFAs uptake fluorescence imaging

Mice (3 animals per group) were injected intraperitoneally with 80 mg/kg body weight of sodium pentobarbital. After anesthesia, animals were injected with 1 μg/g body weight BODIPY-FFA (D3821, Thermo Fisher, MA, USA) via the tail vein, 60-70 min later, the hearts were collected after removing fat and blood and rinsed in PBS. X-ray and fluorescence imaging were performed using a small animal living fluorescence imaging system (Xtrem, Bruker, Germany), and the mean photons was calculated using analysis software corresponding to the imaging systems. The detection parameters are as follows: excitation wavelength 460 nm, emission wavelength 535 nm and exposure time 2 s.

### Histological staining

Histological staining was performed with paraffin sections and frozen sections. For the paraffin sections, the hearts for histological analysis were fixed in 4% paraformaldehyde (pH 7.4) overnight, embedded in paraffin, and serially sectioned to 5 μm thickness. For the dewaxing process, the paraffin sections were placed at 60 °C for 1 h and then transferred to xylene (10 minutes, twice), anhydrous ethanol (3 min, once), 95% ethanol (1 min, once), 70% ethanol (1 min, once), and distilled water (2 min, once). Then, standard hematoxylin and eosin (HE) staining, Masson's staining, Sirius red staining and periodic acid Schiff (PAS) staining were performed as described in previous studies 28-32. After staining, the sections were dehydrated in 95% ethanol (2-3 sec, once), anhydrous ethanol (5-10 sec, twice), and xylene (2 min, twice), and finally sealed with gelatin and photographed with a microscope (DFC700T, Leica, Germany). For the frozen sections, the heart tissues were fixed in 4% paraformaldehyde (pH 7.4) for 24 h, dehydrated with a sucrose gradient, embedded in Tissue-Tek OCT compound (Sakura Finetek, Tokyo, Japan), and serially sectioned at 5 μm. Then, the sections were stained with oil red O (Sigma-Aldrich, St. Louis, MO, USA) for 10 min as described in previous studies and finally sealed with glycerin and photographed with a microscope (DFC700T, Leica, Germany) 29,31. For all histological staining analyses, the positive area was quantified with the ImageJ software program (National Institutes of Health, Bethesda, MD, USA).

### Immunofluorescence

The heart tissues were fixed in 4% paraformaldehyde (pH 7.4) for 24 h, dehydrated with a sucrose gradient, and embedded in the Tissue-Tek OCT compound (Sakura Finetek, Tokyo, Japan). Then, the sections (5 μm) were blocking with 1% bovine serum albumin (Sigma-Aldrich, St. Louis, MO, USA) dissolved in PBS for 60 min at room temperature, and incubated overnight in blocking solution containing antibody against CD31. A second antibody was applied to detect the CD31 protein. PBS was used instead of primary antibody in negative control group, and large blood vessel staining in myocardial tissue sections was used as a positive control. The antibody is listed in Table S1. After the significant CD31-labeled large vessels were excluded, the number of CD31-positive microvessels was counted, and the ratio of microvessel number in diabetic group to that in the control group was calculated. The data analysis was based on three individual mice per group.

### Transmission electron microscopy (TEM)

Cardiac ultrastructure was examined under a transmission electron microscope (JEM-1400, Jeol, Tokyo, Japan) using conventional methods. In brief, heart tissues were fixed with 2.5% glutaraldehyde in 0.1 mol/L phosphate buffer (pH 7.4), followed by 1% OsO4. After dehydration, thin sections were stained with uranyl acetate and lead citrate for observation, images were acquired digitally.

### Dihydroethidium (DHE) staining

Reactive oxygen species (ROS) were measured with DHE staining following incubation at 37 °C for 30 min (Beyotime, Shanghai, China). PBS was used instead of DHE in the negative control group and acetaminophen-induced liver tissue sections were used as the positive control 33. The fluorescence intensity was quantified with the ImageJ software program (National Institutes of Health, Bethesda, MD, USA). The data analysis was based on three individual mice per group.

### Terminal deoxynucleotidyl transferase mediated dUTP nick end labeling (TUNEL) staining

The hearts were fixed in 4% paraformaldehyde (pH 7.4) overnight, embedded in paraffin, and serially sectioned at 5 μm. Sections were deparaffinized and hydrated in xylene and gradient concentrations of ethanol, then incubated in proteinase K at room temperature for 30 min and stained with TUNEL kit (Sigma-Aldrich, St. Louis, MO, USA). Label solution was used instead of TUNEL reagent in the negative control group. All the images were captured by a fluorescence microscope (DFC700T, Leica, Germany). Cells that were positive for TUNEL staining and aligned with DAPI staining were considered apoptotic cells and counted. The number of apoptotic cells in the diabetic group and control group was calculated. The data analysis was based on three individual mice per group.

### Quantitative real-time polymerase chain reaction (Q-PCR)

Total RNA was isolated from the heart tissues using the RNAiso Plus according to the manufacturer's instruction (Takara, Tokyo , Japan). Five hundred nanograms of total RNA was reverse transcribed into complementary DNA in a 10 μl reaction mixture using the PrimeScriptTM RT reagent kit (Takara, Tokyo, Japan). The Q-PCR was performed using the SYBR Green PCR Master Mix (Takara, Tokyo, Japan) and the ABI Q6 Flex Real-Time PCR machine (Applied Biosystems, Foster City, CA, USA). The relative gene expression levels were analyzed using the 2(-ΔΔCt) method and normalized against GAPDH expression. The primers used are listed in Table S2.

### RNA-seq

Total RNA was extracted from hearts of *db/db* mice at 24th week of course, STZ induced mice at 36th week of course, and corresponding control mice (3 samples per group). RNA sequence was performed with BGISEQ-500 platform. In brief, the first step in the workflow involved purifying the poly-A containing mRNA molecules using poly-T oligo-attached magnetic beads. Following purification, the mRNA was fragmented into small pieces using divalent cations under elevated temperature. The cleaved RNA fragments were copied into first strand cDNA using reverse transcriptase and random primers. This was followed by second strand cDNA synthesis using DNA Polymerase I and RNase H. These cDNA fragments then had the addition of a single 'A' base and subsequent ligation of the adapter. The products were then purified and enriched with PCR amplification. We then quantified the PCR yield by Qubit and pooled samples together to make a single strand DNA circle (ssDNA circle), which gave the final library. DNA nanoballs (DNBs) were generated with the ssDNA circle by rolling circle replication (RCR) to enlarge the fluorescent signals at the sequencing process. The DNBs were loaded into the patterned nanoarrays and single-end read of 50 bp were read through on the BGISEQ-500 platform for the following data analysis study. For this step, the BGISEQ-500 platform combined the DNA nanoball-based nanoarrays and stepwise sequencing using Combinational Probe-Anchor Synthesis Sequencing Method. Tools such as HISAT2, Bowtie2, Cluster *etc.* were used for the following bioinformatics analysis. In the analysis of differentially expressed genes (DEGs), the genes that had more than double fold change and the corrected P value is less than or equal to 0.05 were defined as DEGs. With DEGs, we performed KEGG pathway classification and functional enrichment using R. To ensure the accuracy of the bioinformatics analysis, data from only 9 well-relevant samples entered the final bioinformatics analysis.

### Western blotting analysis

The heart tissues were lysed with RIPA buffer (Millipore, Bedford, MA, USA) for total protein extraction. Nuclear proteins were extracted using a nuclear protein extraction kit (Beyotime, Shanghai, China). The protein concentration was determined using the BCA protein assay kit (Millipore, Bedford, MA, USA) according to the manufacturer's protocol. Equal amounts of protein (30 μg) were subjected to SDS-PAGE for electrophoresis, transferred to 0.45 μm PVDF membranes (Millipore, Bedford, MA, USA), and immunoblotted with antibodies. The bands were quantified using the ImageJ software program (National Institutes of Health, Bethesda, MD, USA). The antibodies are listed in Table S1.

### Human heart samples

Twenty-eight consecutive human heart samples were collected from decedents with clear diagnosis from January 5, 2017 to May 2, 2018 at the National Center for Medico-legal Expertise of Sun Yat-sen University. All sample donors were clearly diagnosed with T2DM but without coronary heart disease and hypertension. Since the body weight index was missing in the forensic examination, the BMI of the donors was unknown. According to previous studies, we defined an obese population as individuals with an abdominal fat thickness greater than or equal to 2.74 cm and a non-obese population as individuals with an abdominal fat thickness less than 2.74 cm 34,35. Based on the above criteria, three heart samples from nondiabetic patients were excluded because their donors had abdominal fat thicker than 2.74 cm; after these samples were removed, twenty-five heart samples were used in this study. The samples were categorized into four groups as follows. Group one comprised six samples from a healthy population of gender-age matched individuals that served as controls for T2DM patients with obesity; group two comprised six samples of T2DM patients with obesity; group three comprised eight samples from a healthy population of gender-age matched individuals that served as controls for T2DM patients with non-obesity; and group four comprised eight samples from T2DM patients with non-obesity. Three heart samples were categorized as being in group one and group three. Information of these samples were provided in the Tables S3. This study was approved for human research by the ethics committee of Sun Yat-sen University and all data and sample collection were in strict accordance with ethics guidelines of Zhongshan School of Medicine, Sun Yat-sen University. Informed consent was obtained from the legal representatives of the victims. The principles outlined in the Declaration of Helsinki were followed.

### Immunohistochemistry (IHC)

Human heart samples were fixed in 4% paraformaldehyde overnight followed by conventional dehydration and slicing. Then, the human heart samples sections (5 μm) were blocked with 3% hydrogen peroxide and then performed at 95 °C for 10 min using citrate buffer (Beyotime, Shanghai, China), then blocking steps were carried out using the QuickBlock™ Blocking Buffer (Beyotime, Shanghai, China) according to the manufacturer's instructions. After incubated with primary antibody at 4 °C overnight, the sections incubated with secondary antibody (G1210, Servicebio, Wuhan, Hubei, China) at 37 °C for 30 min. Visualization was accomplished using 3,3N-diaminobenzidine tertrahydrochloride (G1211, Servicebio, Wuhan, Hubei, China). Sections were counterstained with hematoxylin (Servicebio, Wuhan, Hubei, China). Rabbit IgG (2729S, Cell signaling technology, Beverly, MA, USA) was used instead of a primary antibody in the negative control group. The mean density was quantified from 4-6 fields per sample with the Image-pro plus software program (Media Cybernetics, Rockville, MD, USA). The antibodies are listed in Table S1.

### Statistical analysis

All analyses were performed with the Statistical Package for Social Sciences version 19.0 (SPSS, Chicago. IL, USA). The data were expressed as the mean ± SD. Statistical differences between two groups were analyzed by the unpaired Student's t test and differences between multiple groups of data were analyzed by one-way ANOVA. In all statistical comparisons, a p value <0.05 was used to indicate a statistically significant difference.

## Results

### Both the obese *db/db* and the non-obese STZ-induced mouse models have a T2DM phenotype

Since *db/db* mice develop stable hyperglycemia and hyperinsulinemia at 8 weeks of age, we used 8-week-old *db/db* mice to define the baseline for the duration of diabetes mellitus. Our experiments lasted 24 weeks after the onset of diabetes (Figure S1A). During the entire observational period, compared to the *db/m* mice, the *db/db* mice exhibited persistent obesity, hyperglycemia and hyperinsulinemia with a higher HOMA-IR 4, 12 and 24 weeks after disease onset (Figure S1B-E). The results from the ITT assay showed that the blood glucose of the *db/db* mice did not change after the insulin injection 4 weeks after disease onset, leading to a larger AUC than that for the *db/m* mice (Figure S1F).

Correspondingly, the low-dose STZ injection combined with HFD-induced diabetes mellitus led to a stably hyperglycemic phenotype 2 weeks after the final STZ injection. Our observations extended from this time point until the 36th week of the disease (Figure S1G). Compared with the control group, the diabetes mellitus (DM) group was persistently non-obese and exhibited hyperglycemia and hyperinsulinemia, with a higher HOMA-IR at 4, 12 and 24 weeks of the disease (Figure S1H-K). However, after 36 weeks of the disease, the DM mice exhibited significantly decreased serum insulin levels compared with that of the control mice, possibly because of long-term insulin resistance (Figure S1J). Notably, although the DM mice exhibited significantly decreased blood glucose at 36 weeks compared with the level measured at the 32nd week, the blood glucose level remained higher than it was for the control group, which had a normal HOMA-IR (Figure S1I and K). Moreover, the GTT and ITT data revealed that the DM mice had a lower tolerance to glucose and insulin at 4 or 24 weeks, leading to a larger AUC than that for the control mice (Figure S1L and M). HE-stained samples revealed that the islets of the diabetic mice did not exhibit significant damage at 24 weeks but were completely destroyed at 36 weeks because of long-term insulin resistance (Figure S1N).

### Obese T2DM mice exhibit more severe cardiac structure remodeling and faster cardiac systolic dysfunction than non-obese T2DM mice

**Figure 2:**
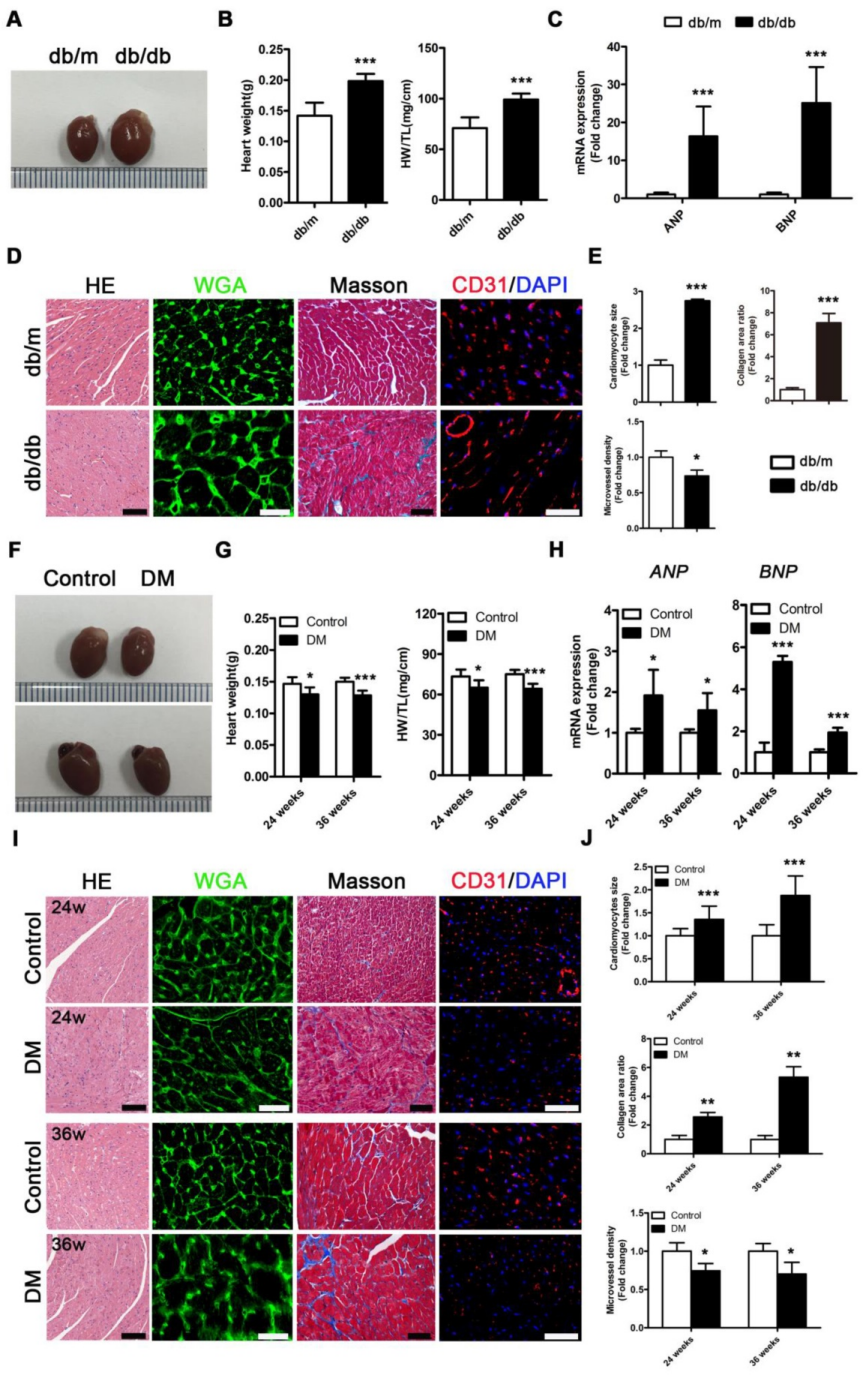
** Cardiac structure of obese and non-obese T2DM mice.** (A) Macroscopic pictures of hearts from obese T2DM mice in the 24th week of the disease course. (B) Heart weight and heart weight/tibia length ratio from obese T2DM mice in the 24th week of the disease course. (n = 6) (C) Fold change of mRNA expression levels of ANP and BNP in heart tissues from obese T2DM mice in the 24th week of the disease course. (n = 4-6) (D) HE staining (Scale bar = 100 μm), WGA staining (Scale bar = 30 μm), Masson staining (Scale bar = 50 μm) and CD31 immunofluorescence staining (Scale bar = 100 μm) of heart tissues from obese T2DM mice in the 24th week of the disease course. (E) Changes in cardiomyocyte size, collagen area ratio and microvessel density based on panel D. (n = 3) (F) Macroscopic pictures of hearts from non-obese T2DM mice in the 24th and 36th weeks of the disease course. (G) Heart weight and heart weight/tibia length ratio from non-obese T2DM mice in the 24th and 36th weeks of the disease course. (n = 6) (H) Fold change of mRNA expression levels of ANP and BNP in heart tissues from non-obese T2DM mic**e** in the 24th and 36th weeks of the disease course. (n = 4-6) (I) HE staining (Scale bar = 100 μm), WGA staining (Scale bar = 30 μm), Masson staining (Scale bar = 50 μm) and CD31 immunofluorescence staining (Scale bar = 100 μm) of heart tissues from non-obese T2DM mice in the 24th and 36th weeks of the disease course. (J) Changes in cardiomyocyte size, collagen area ratio and microvessel density based on panel I. (n = 3) Data are expressed as mean ± SD. (*, p<0.05; **, p<0.01; ***, p<0.001).

*Db/db* mice exhibited a typical DCM phenotype at the 24th week of the disease that was primarily characterized by enlarged ventricles, increased cardiac weight and heart/tibia ratio, upregulated atrial natriuretic peptide (ANP) and brain natriuretic peptide (BNP) levels, overt cardiomyocyte hypertrophy, myocardial fibrosis and decreased microvessel density (Figure 2A-E). However, in contrast to *db/db* mice, the non-obese T2DM mice had smaller ventricles than the control group, and these mice exhibited decreased cardiac weight and heart/tibia ratio at the 24th and 36th week of diabetes (Figure 2F and G). Correspondingly, the non-obese T2DM mice also exhibited the formation of cardiac remodeling, which featured upregulated ANP and BNP, overt cardiomyocyte hypertrophy, myocardial fibrosis, and decreased microvessel density at the 24th and 36th weeks of diabetes (Figure 2H-J).

**Figure 3:**
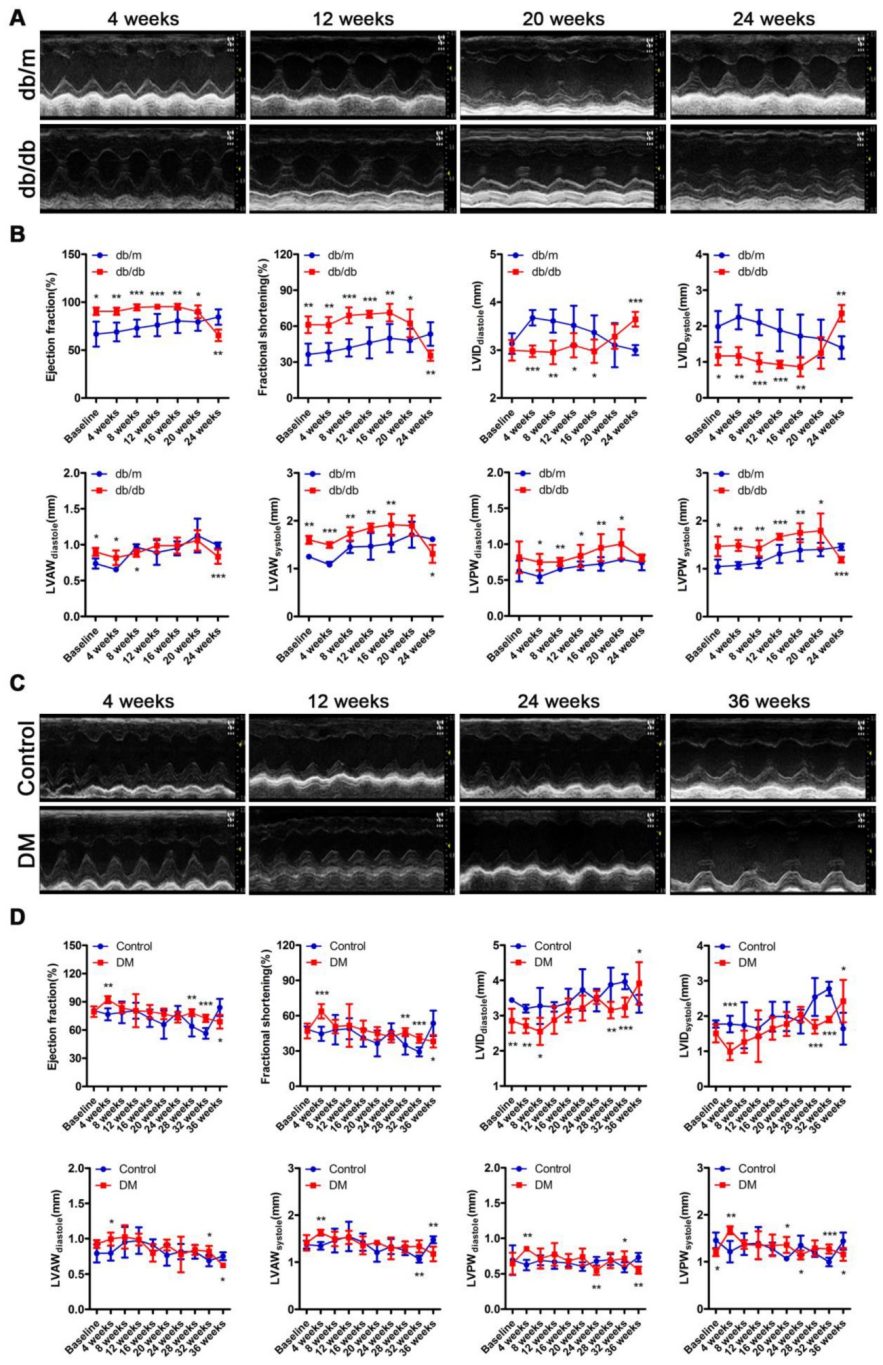
** Cardiac function of obese and non-obese T2DM mice.** (A) Echocardiography of obese T2DM mice. (B) Data related to cardiac function of obese T2DM mice. (n = 6-10) (C) Echocardiography of non-obese T2DM mice. (D) Data related to cardiac function of non-obese T2DM mice. (n = 6-10) Data are expressed as mean ± SD. (*, p<0.05; **, p<0.01; ***, p < 0.001).

The cardiac function of the *db/db* mice was in a compensatory period from 0 to 20 weeks of the disease, which was characterized by an increased ejection fraction (EF) and fractional shortening (FS), a decreased left ventricular internal diameter (LVID) at the end of diastole and end of systole, and a thicker left ventricular posterior wall (LVPW) and left ventricular anterior wall (LVAW) at the end of diastole and end of systole (Figure 3A and B). However, the cardiac function of the *db/db* mice shifted from a compensatory stage to a decompensatory stage at the 24th week of diabetes and exhibited systolic dysfunction (Figure 3A and B). Moreover, the echocardiography results showed that the non-obese T2DM mice were in the cardiac function compensatory stage at the 4th, 28th and 32nd week of the disease, and these mice exhibited systolic dysfunction at the 36th week (Figure 3C and D).

In addition, although the heart rate, aortic ejection time (AET) and isovolumetric contraction time (IVCT) did not change, impaired diastolic function characterized by decreased E/A ratio and prolonged isovolumic relaxation time (IVRT) were found in the* db/db* mice through the results of the mitral valve rheography testing (Figure S2A-F). Moreover, although the E/A ratio of DM mice did not change, the mouse heart rate decreased, and diastolic function was impaired, as indicated by the prolonged AET, IVCT and IVRT on mitral valve rheography at the 24th or/and 36th week (Figure S2G-L).

### Severe myocardial lipotoxicity is found in obese T2DM mice but not in non-obese mice with cardiac systolic dysfunction

**Figure 4:**
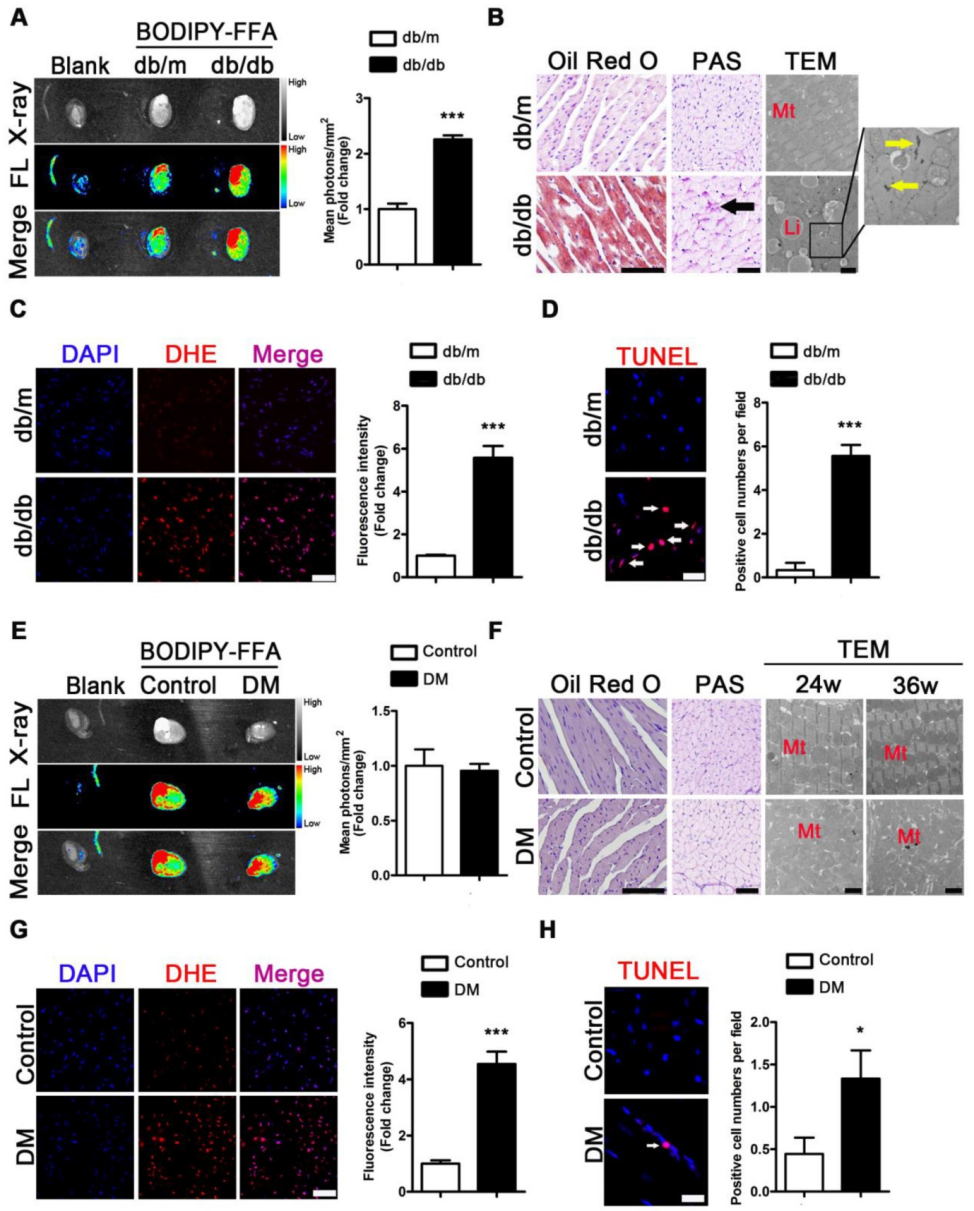
** Metabolic situation in hearts of obese and non-obese T2DM mice.** (A) Fluorescence imaging of cardiac FFAs uptake from obese T2DM mice in the 24th week of the disease course and mean photons per square millimeter calculated. (n = 3) (B) Oil red O staining (Scale bar = 100 μm), PAS staining (Scale bar = 50 μm; Black arrow indicates glycogen particles) and TEM pictures (Scale bar = 2 μm; Mt, mitochondria; Li, lipid droplet; Yellow arrow indicates glycogen particles) of heart tissues from obese T2DM mice in the 24th week of the disease course. (C) DHE staining of heart tissues from obese T2DM mice in the 24th week of the disease course (Scale bar = 50 μm) and the fluorescence intensity. (n = 3) (D) TUNEL staining of heart tissues from obese T2DM mice in the 24th week of the disease course (Scale bar = 20 μm) and the number of apoptotic cells. (n = 3) (E) Fluorescence imaging of cardiac FFAs uptake from non-obese T2DM mice in the 36th week of the disease course and mean photons per square millimeter calculated. (n = 3) (F) Oil red O staining (Scale bar = 100 μm), PAS staining (Scale bar = 50 μm; Black arrow indicates glycogen particles) and TEM pictures (Scale bar=2 μm; Mt, mitochondria; Li, lipid droplet; Yellow arrow indicates glycogen particles) of heart tissues from non-obese T2DM mice in the 24th and/or 36th week of the disease course. (G) DHE staining of heart tissues from non-obese T2DM mice in the 36th week of the disease course (Scale bar = 50 μm) and the fluorescence intensity. (n = 3) (H) TUNEL staining of heart tissues from non-obese T2DM mice in the 36th week of the disease course (Scale bar = 20 μm) and the number of apoptotic cells. (n = 3) Data are expressed as mean ± SD. (*, p <0.05; ***, p<0.001.).

To evaluate the metabolic adaptations in energy by the T2DM mice, we measured the serum lipid content. Increased FFAs, TG, TC, and LDL as well as decreased HDL levels were observed in the sera of the *db/db* mice compared with the sera of the *db/m* mice (Figure S3A-E). To investigate the FFAs uptake in the hearts of obese diabetic mice, we used fluorescence imaging with mice administered BODIPY-FFA via tail vein injection at a dose of 1 μg/g body weight. The fluorescence images of the tissues revealed that the hearts of the *db/db* mice had greater FFAs uptake than those of the *db/m* mice (Figure 4A). Furthermore, the histopathological and TEM results revealed an abundance of lipid droplets and glycogen present in the hearts of the *db/db* mice but not in the hearts of *db/m* mice (Figure 4B).

The same test was performed for the mice of the DM group and the corresponding control group. Although increased FFAs, TG, TC, and LDL and decreased HDL levels in serum were observed in the DM mice compared with the control mice (Figure S3F-J), fluorescence imaging showed no differences in BODIPY-FFA uptake between the hearts of the DM and control mice (Figure 4E). Moreover, the histological data revealed no significant lipid droplets or glycogen deposition in the hearts of the DM mice (Figure 4F). In addition, although neither lipid droplets nor glycogen deposits were observed by TEM, significant mitochondrial hyperplasia was observed at the 24th and 36th weeks of the disease (Figure 4F).

Because oxidative stress and apoptosis are important pathological mechanisms of DCM and are subsequent reactions to lipotoxicity, we performed the corresponding assessments. DHE-stained samples showed significantly increased accumulation of superoxide anion radicals in the hearts of the *db/db* and DM mice compared to that in the hearts of the *db/m* and control mice (Figure 4C and G). In addition, TUNEL-stained apoptotic cells were observed in the hearts of obese diabetic mice at the 24th week of the disease (Figure 4D). The same results were observed at the 36th week of the disease in the hearts of the DM mice (Figure 4H).

### RNA sequencing reveals that activation of the PPARα pathway may be a key factor leading to myocardial lipotoxicity in obese T2DM mice

**Figure 5:**
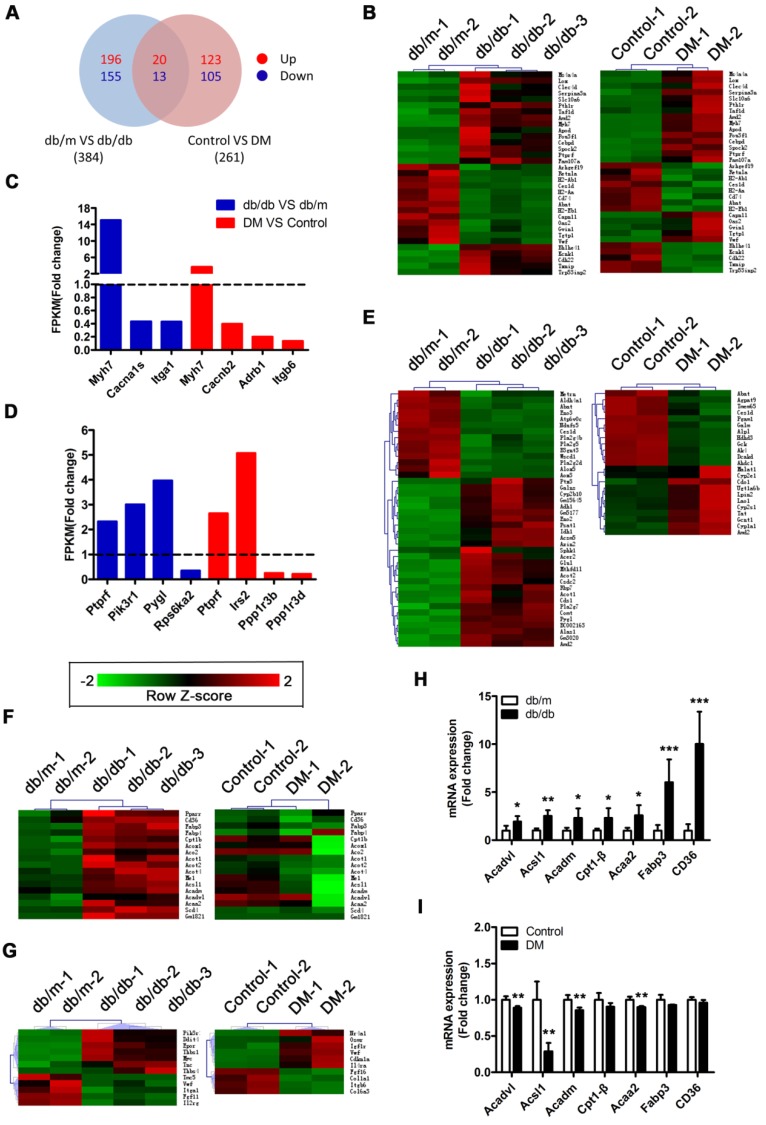
** RNA sequencing results.** (A) Venn diagram of DEGs. (B) Common DEGs shared by the two models. (C) DEGs related to cardiomyopathy of the two models. (D) DEGs related to insulin resistance of the two models. (E) DEGs related to the metabolic pathway of the two models. (F) DEGs related to the PPAR pathway of the two models. (G) DEGs related to the PI3K/Akt pathway of the two models. (H) Q-PCR of genes related to the PPARα pathway in obese T2DM mice. (n = 4-6) (I) Q-PCR of genes related to the PPARα pathway in non-obese T2DM mice. (n = 3-4) Data are expressed as mean ± SD. (*, p<0.05; **, p<0.01; ***, p < 0.001).

A Venn diagram showed that the two mouse models shared a total of 33 differentially expressed genes (DEGs), including the *protein tyrosine phosphatase receptor type f (Ptprf)* gene, which is associated with insulin resistance, and the *beta-myosin heavy chain gene (Myh7)* gene, which is associated with cardiomyopathy (Figure 5A and B). In addition, the two models also expressed unique genes related to insulin resistance and cardiomyopathy (Figure 5C and D). This result suggested that both models exhibited insulin resistance and cardiomyopathy but that the underlying pathological mechanisms differed. Importantly, we determined that 41 DEGs associated with metabolic pathways were enriched in the *db/db* mice, whereas only 23 DEGs associated with metabolic pathways were enriched in the DM mice (Figure 5E). This finding suggested that the hearts of the *db/db* mice exhibited more severe metabolic changes. Furthermore, 17 genes associated with the peroxisome proliferator activated receptor (PPAR) pathway were significantly changed in the hearts of the *db/db* mice, whereas no significant expression changes in the genes associated with this pathway were observed in the hearts of the DM mice (Figure 5F). Since the relationship between PPARα and myocardial lipid metabolism has been well established through studies and because multiple downstream target genes of PPARα, such as fatty acid-binding protein 3 (FABP3) and fatty acid translocase/CD36 (FAT/CD36), were screened out of the analysis, this result indicates that the PPARα pathway may be the key factor leading to the differences observed between the two models 36. Moreover, we determined that 12 and 10 protein kinase B (Akt) pathway-related genes were enriched in the hearts of the *db/db* mice and DM mice, respectively; however, only one DEG was shared, and the change trend for that gene was opposite in the models (Figure 5G). This finding suggests that the Akt pathway may also play a key role in the differences observed between the two models. To verify the accuracy of the RNA sequences, we selected 7 PPARα pathway-related genes for Q-PCR validation. The results revealed that these 7 genes were significantly upregulated in the hearts of the *db/db* mice but not in those of the DM mice, a finding that was consistent with the sequencing results (Figure 5H and I).

### Proteins related to lipotoxicity are upregulated throughout the course of T2DM in obese mice but only in the early stages of T2DM in non-obese mice

**Figure 6:**
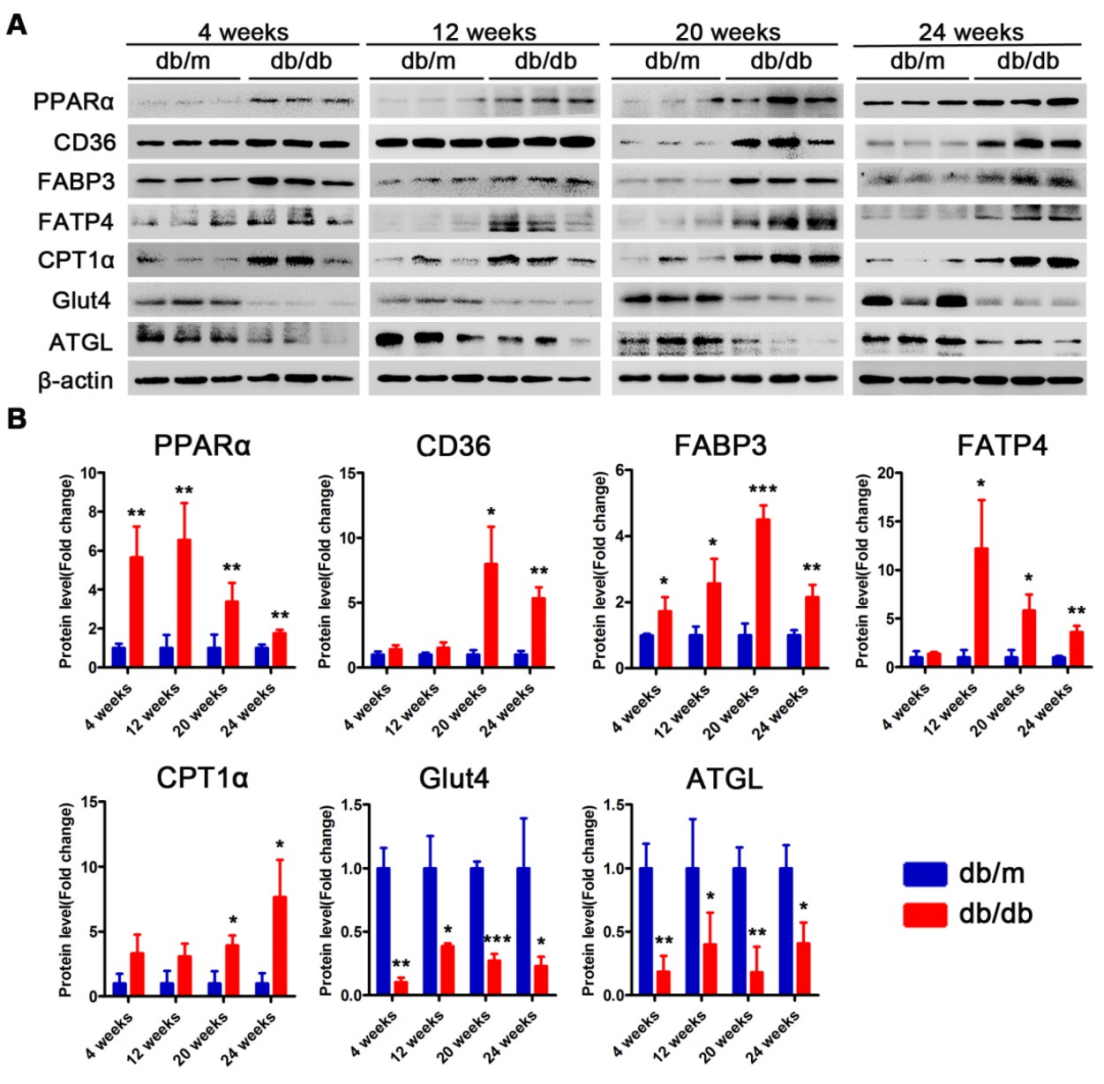
** Western blot results of proteins related to energy metabolism in heart tissues of obese T2DM mice.** (A) Western blot images of proteins related to energy metabolism in heart tissues from obese T2DM mice. (B) Relative quantification based on the results of panel A. (n = 3) Data are expressed as mean ± SD. (*, p<0.05; **, p<0.01; ***, p < 0.001).

**Figure 7:**
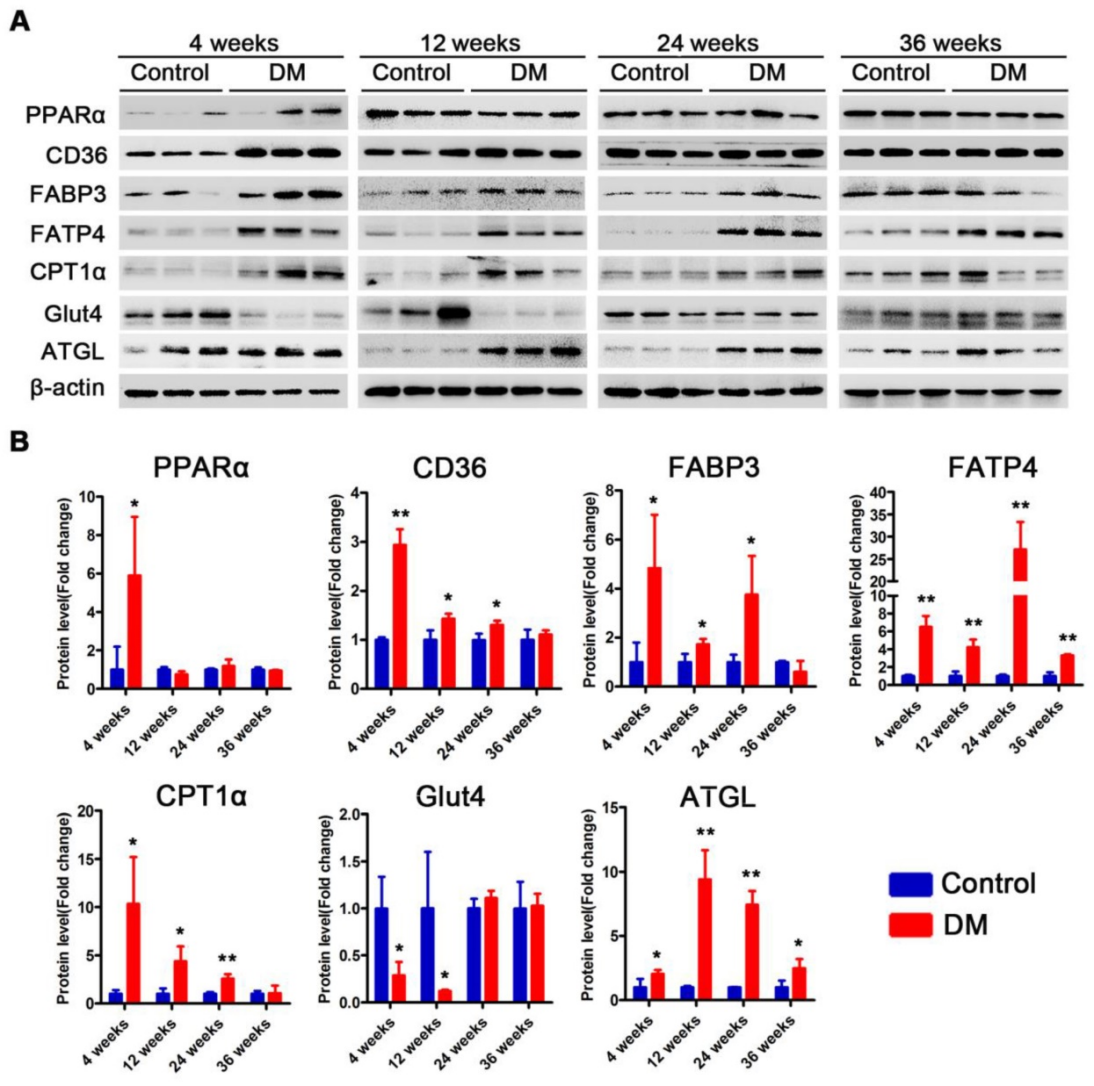
** Western blot results of proteins related to energy metabolism in heart tissues of non-obese T2DM mice.** (A) Western blot images of proteins related to energy metabolism in heart tissues from non-obese T2DM mice. (B) Relative quantification based on the results of panel C. (n = 3-6) Data are expressed as mean ± SD. (*, p<0.05; **, p<0.01; ***, p < 0.001).

Subsequently, we examined changes in the proteins related to lipid metabolism and glucose metabolism at the 4th, 12th and 24th week of diabetes mellitus in the *db/db* mice and at the 4th, 12th, 24th and 36th week of diabetes mellitus in the DM mice. The protein expression levels of PPARα, CD36, FABP3, fatty acid transporter 4 (FATP4) and carnitine acyltransferase 1 alpha (CPT1α), which are related to FFAs uptake and oxidation, were significantly upregulated in the hearts of the *db/db* mice compared with those of the *db/m* mice throughout the course of diabetes, whereas the expression of adipose triglyceride lipase (ATGL), which is an enzyme that promotes triglyceride hydrolysis, was significantly downregulated throughout the course of the disease (Figure 6A and B). In addition, glucose transport protein 4 (GLUT4) expression was significantly decreased in the hearts of the *db/db* mice compared with the hearts of the *db/m* mice throughout the course of the disease (Figure 6A and B). Correspondingly, the expression of proteins related to FFAs uptake and oxidation was significantly upregulated in the hearts of the DM mice in the early stages of diabetes but gradually returned to normal in the later stages of diabetes (Figure 7A and B). However, ATGL expression was significantly upregulated in the hearts of the DM mice compared with the hearts of the control mice at both the early and late stages of diabetes (Figure 7A and B). In addition, GLUT4 expression was significantly decreased in the hearts of the DM mice compared with the hearts of the control mice in the early stages of diabetes but gradually returned to normal in the late stages of diabetes (Figure 7A and B).

### Glycogen synthesis-related molecules are continuously activated in obese T2DM mice but are continuously inhibited in non-obese T2DM mice

Subsequently, we examined changes in the proteins that were related to insulin resistance and glycogen synthesis at the 4th, 12th and 24th weeks of diabetes mellitus in *db/db* mice and at the 4th, 12th, 24th and 36th weeks of diabetes mellitus in DM mice. The Akt pathway was suppressed in the hearts of *db/db* mice, as manifested by Akt dephosphorylation; however, phosphorylated glycogen synthase kinase 3 beta (p-GSK-3β) and total glycogen synthase kinase 3 beta (t-GSK-3β) expression were significantly increased (Figure S4A and B). Correspondingly, the Akt pathway was suppressed in the hearts of DM mice, as manifested by Akt dephosphorylation; however, unlike in the *db/db* mice, p-GSK-3β and total GSK-3β expression were significantly decreased (Figure S4C and D).

### The anti-oxidative pathway is significantly activated during the systolic dysfunction phase in obese T2DM mice but is significantly inhibited in non-obese T2DM mice

Subsequently, we examined changes in the proteins related to oxidative stress and apoptosis at the 4th, 12th and 24th week of diabetes mellitus in the *db/db* mice and at the 4th, 12th, 24th and 36th week of diabetes mellitus in the DM mice. The expression of the transcription factor NF-E2-related factor 2 (Nrf2) in the nucleus and its target genes heme oxygenase-1 (HO-1) and NAD(P)H:quinone oxidoreductase 1 (NQO1) at the 20th and 24th weeks of diabetes was significantly increased in the *db/db* mice compared with the *db/m* mice (Figure S5A and B). In the hearts of DM mice, the expression of the transcription factor Nrf2 and its target genes was not obviously changed at the 4th, 12th and 24th week; however, the levels of these proteins were significantly decreased at the 36th week of diabetes compared with those in the control mice (Figure S6A and B). In addition, both Bax expression and the cleaved-caspase-3/caspase-3 expression ratios were significantly increased in the hearts of the *db/db* mice and DM mice, whereas B-cell lymphoma-2 (Bcl-2) expression was significantly decreased in the hearts of both diabetic models (Figure S5A and B, Figure S6A and B).

### Myocardial injury is more severe in obese T2DM patients than in non-obese T2DM patients

**Figure 8:**
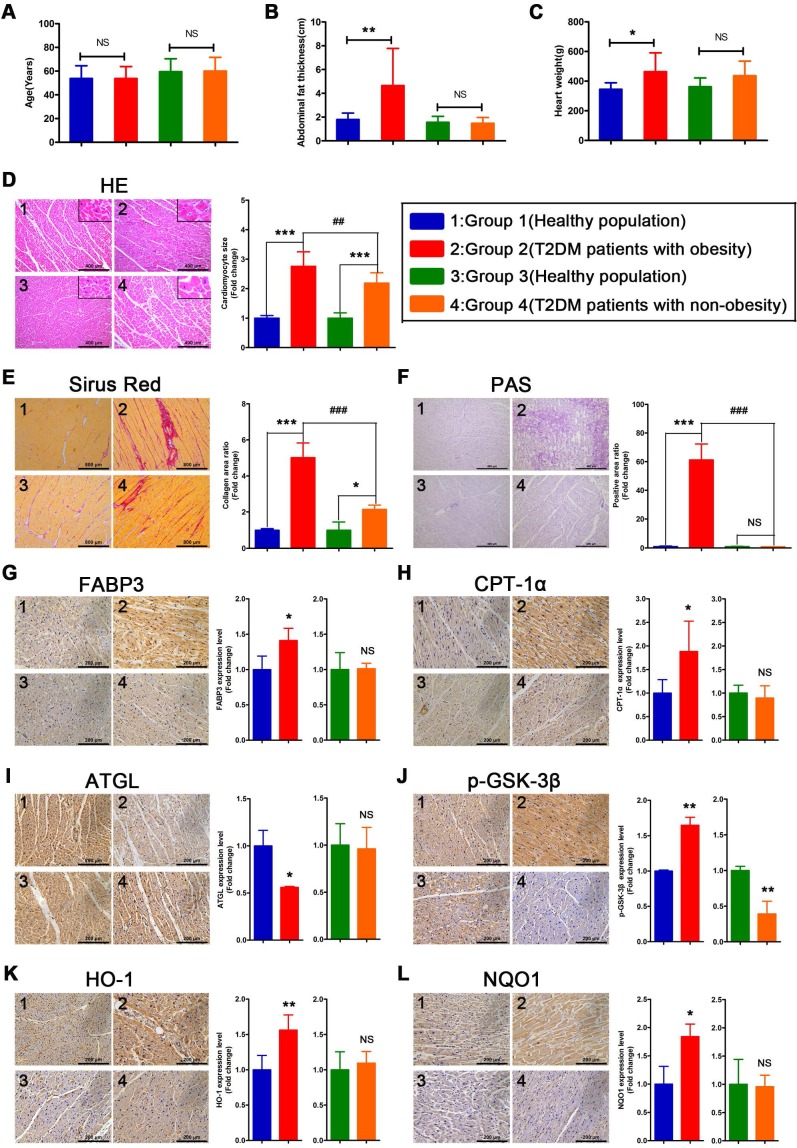
** The information of donors, histological results and IHC results in human heart tissues.** (A) Age of donors. (n = 6-8) (B) Abdominal fat thickness of donors. (n = 6-8) (C) Heart weight of donors. (n = 6-8) (D) HE staining and cardiomyocyte size. (n = 6-8; Scale bar = 400 μm) (E) Sirus red staining and collagen area ratio. (n = 3; Scale bar = 800 μm) (F) PAS staining and positive area ratio. (n = 3; Scale bar = 400 μm) (G) IHC images and relative quantification of FABP3. (n = 4-5; Scale bar = 200 μm) (H) IHC images and relative quantification of CPT-1α. (n = 4-7; Scale bar = 200 μm) (I) IHC images and relative quantification of ATGL. (n = 3; Scale bar = 200 μm) (J) IHC images and relative quantification of p-GSK-3β. (n = 3-4; Scale bar = 200 μm) (K) IHC images and relative quantification of HO-1. (n = 5-8; Scale bar = 200 μm) (L) IHC images and relative quantification of NQO1. (n = 3-4; Scale bar = 200 μm) Data are expressed as mean ± SD. (*, p<0.05; **, p<0.01; ***, p<0.001; ##, p<0.01; ###, p<0.001.)

To verify whether the relevant phenotypes found in mouse models could be verified in humans, the corresponding tests performed on mice samples were performed on human heart samples. Since heart samples from diabetic patients with systolic dysfunction or heart failure are difficult to obtain, we collected cardiac tissue from non-heart-failure diabetic patients to verify the relevant mechanisms. The results revealed no significant differences in age between the four groups of donors (Figure 8A). In addition, we determined that the obese T2DM patients exhibited not only greater abdominal fat thickness but also greater heart weight than the healthy population (Figure 8B and C). In addition, the abdominal fat thickness and heart weight of the non-obese T2DM patients did not differ significantly from those of the healthy population (Figure 8B and C). Moreover, the HE- and Sirius red-stained samples indicated that both obese and non-obese T2DM patients exhibited severe cardiac hypertrophy and myocardial fibrosis, and the obese T2DM patients exhibited more severe cardiac remodeling than the non-obese T2DM patients (Figure 8D and E). The PAS-stained samples showed that a large amount of glycogen had been deposited in the hearts of the obese T2DM patients but not in the hearts of the non-obese T2DM patients or in the hearts from the healthy individuals (Figure 8F).

### Severe myocardial lipotoxicity and activated glycogen synthesis-related molecules and antioxidative pathways are found in obese T2DM patients but not in non-obese T2DM patients

To validate the relevant mechanisms, we further examined the expression of proteins involved in lipid metabolism, glycogen synthesis, and oxidative stress. The IHC results revealed that the expression levels of FABP3 and CPT-1α were significantly upregulated in the heart tissues of the obese T2DM patients compared with those of the healthy individuals, but the expression of these proteins was not upregulated in the non-obese T2DM patients (Figure 8G and H). Additionally, the expression of ATGL was significantly downregulated in the heart tissues of the obese T2DM patients compared with the tissues of the healthy individuals but was not obviously different between the heart tissues of the non-obese T2DM patients and the healthy individuals. (Figure 8I) The expression of p-GSK-3β in heart tissue was significantly upregulated in the obese T2DM patients but significantly downregulated in the non-obese T2DM patients compared with the healthy individuals (Figure 8J). Moreover, the expression of HO-1 and NQO1 in heart tissue was significantly upregulated in the obese T2DM patients but was not obviously different in the non-obese T2DM patients compared with the healthy individuals (Figure 8K and L).

## Discussion

In this study, two animal models of T2DM were successfully induced. Between them, the *db/db* mice exhibited the phenotype of obese T2DM, and low-dose STZ injection combined with HFD-induced diabetes resulted in the phenotype of non-obese T2DM. It is important to note that the STZ-induced T2DM mice remain non-obese or even thin when fed a HFD, which is pattern consistent with a portion of the results of previous studies and is likely due to metabolic imbalance and/or partial islet destruction and may be associated with end-stage eating disorder 37-40. The group of DM mice exhibited a significant reduction in serum insulin levels at the 36th week, possibly due to long-term insulin resistance, which can undermine the secretory function of islet β cells. Both T2DM models exhibited a DCM phenotype that featured cardiomyocyte hypertrophy, myocardial fibrosis, decreased microvessel density, and upregulated ANP and BNP expression; however, the obese T2DM mice exhibited more severe myocardial injury than did the non-obese T2DM mice. It is important to emphasize that, although the two models showed the phenotypes of obese and non-obese T2DM mice consistently, respectively, it cannot be ruled out that the differences in findings are the result of different modeling methods. However, as these are classic animal models, we contend that they provide evidence that can be further verified in human trials.

DCM eventually leads to heart failure, including heart failure with reduced ejection fraction (HFrEF) and heart failure with preserved ejection fraction (HFpEF) 41. In the two models we used, the obese T2DM mice showed a more sustained compensatory period and earlier decompensation that led to early cardiac systolic dysfunction, while the non-obese T2DM mice showed intermittent compensation periods that led to a relatively prolonged time before decompensation and resulted in a decline in systolic function. This finding confirms that obesity is a high-risk factor for heart failure; however, the specific obesity-related mechanism remains ambiguous. HFpEF is now the most common form of heart failure, and the main risk factors for it are obesity and diabetes, with approximately 45% of patients with HFpEF also having diabetes 42. Long-term hyperglycemia can reduce heart compliance in a variety of ways, leading to diastolic dysfunction and even HFpEF, mainly including increased myocardial fibrosis, increased endothelial dysfunction, increased systolic blood pressure, *etc* 42. Interestingly, the obese and non-obese T2DM mice showed different heart rate changes and different diastolic dysfunction phenotypes. The obese T2DM mice showed more typical characteristics, namely, a decrease in the E/A ratio, while the non-obese T2DM mice showed a more specific diastolic dysfunction, mainly manifested by an unchanged E/A ratio and significantly prolonged IVCT and IVRT, which may have been related to their significantly reduced heart rate. The non-obese T2DM mice showed a significant decrease in heart rate during the 36-week course of the disease, which may have been due to insufficient blood supply to the heart or atrioventricular blockages caused by their long-term hyperglycemia, and the decrease in heart rate may have further affected the diastolic function of the mice, especially the prolongation of IVCT and IVRT 43. The above results suggest that, compared to non-obese T2DM mice, obese T2DM mice exhibit typical diastolic dysfunction earlier, and similarly, its pathological mechanism remains unclear. Therefore, finding the pathophysiological differences in myocardial injury between obese and non-obese T2DM and accurately characterizing them has great significance for the accurate diagnosis and treatment of myocardial injury in T2DM patients. Subsequently, we aimed to determine the differences in cardiac energy metabolism and oxidative stress adaptations between the hearts of obese and non-obese diabetic mice.

FFAs are important sources of energy in the body under normal physiological conditions, and 60% to 70% of the energy of cardiomyocytes is derived from the complete oxidation of FFAs 44. However, in the case of obesity, metabolic syndrome, diabetes and other pathological conditions, plasma FFAs increase significantly and persistently. The accumulation of excess FFAs in nonfat cells leads directly to glucose and lipid metabolic disorders, including lipotoxicity and insulin resistance 45,46. In addition, excess FFAs can induce an imbalance in the uptake and utilization of FFAs in cardiomyocytes, resulting in excessive FFAs uptake and oxidation in the heart, which in turn lead to cardiomyocyte damage 47-49. In this study, the obese T2DM mice exhibited severe myocardial lipotoxicity that was characterized by increased FFAs uptake, the accumulation of lipid droplets, glycogen and ROS, whereas no significant differences in lipotoxicity features were observed between the non-obese T2DM mice and the respective control mice.

The transmembrane transport of circulating FFAs into cardiomyocytes is a key step in lipotoxicity damage to the myocardium. Recently, a number of studies have suggested that more than 70% of the FFAs taken up by cardiomyocytes are transported by protein-mediated active transport 50. Three proteins, including FABP3, CD36 and FATP4, are thought to play roles as transport carriers in the transmembrane trafficking of FFAs in cardiomyocytes 50. Among these proteins, CD36 and FABP3 are considered key regulators of FFAs uptake by cardiomyocytes 51. After being transported into cardiomyocytes, FFAs must enter the mitochondria to be oxidized to generate ATP. CPT-1α is the key factor that regulates the intracellular transport of FFAs during this process 52. Furthermore, PPARα is a ligand-activated transcription factor that is related to FFAs uptake and oxidation 36,53. Increased PPARα expression and activity can activate certain target genes, including FABP3, CD36, FATP4 and CPT-1α, thereby enhancing FFAs uptake and oxidation 36. In our study, we discovered that the expression of proteins related to FFAs uptake and oxidation was increased in the hearts of the obese T2DM mice throughout the entire course of the diabetes; however, the expression of these proteins was increased only in the early stage of the disease in the hearts of the non-obese T2DM mice and returned to normal levels in the later stages. The persistent increase in FFAs uptake and oxidation may be responsible for the earlier onset of systolic dysfunction in obese T2DM mice. These results further reveal the pivotal role of lipotoxicity in the differences between the hearts of obese and non-obese T2DM mice. Correspondingly, increased myocardial FFAs uptake and oxidation are often accompanied by decreased glucose uptake in diabetes 54. As GLUT4 is one of the primary regulatory molecules involved in glucose transport into cells, downregulated GLUT4 expression is an important marker of reduced glucose uptake 55. Downregulated GLUT4 expression results in a decrease in glucose uptake by the tissue, resulting in a more dramatic increase in blood glucose levels. In this study, the western blotting results revealed that GLUT4 was continuously downregulated in the hearts of obese T2DM mice, whereas GLUT4 expression in the hearts of the non-obese mice was significantly downregulated in the early stages of the disease and returned to normal in the later stages. These results reveal the differences in energy substrate uptake and utilization in the hearts of the obese and non-obese diabetic mice. In addition, myocardial lipotoxicity can cause a large amount of lipid droplet deposition, and ATGL is the most critical triglyceride hydrolase 56. ATGL expression was continuously downregulated in the hearts of the obese T2DM mice but was continuously upregulated in the hearts of the non-obese T2DM mice, which may explain the abundance of lipid droplets detected in the hearts of the obese T2DM mice but not in the hearts of the non-obese T2DM mice.

As a typical feature of T2DM, insulin resistance often promotes lipotoxicity, creating a vicious circle that exacerbates the development of DCM. The Akt pathway is closely related to insulin resistance and glycogen synthesis, and phosphorylation of Akt activates this pathway 57-59. In the present study, both T2DM models exhibited a phenotype of insulin resistance that was characterized by impaired insulin tolerance or glucose tolerance and reduced Akt phosphorylation. However, a key enzyme for glycogen synthesis, GSK3β (p-GSK3β) was suppressed only in the hearts of the non-obese T2DM mice; in contrast, the expression of GSK3β (p-GSK3β) was increased in the hearts of the obese T2DM mice. This result may potentially explain the accumulation of glycogen detected in the hearts of the obese T2DM mice but not in the non-obese T2DM mice. Importantly, massive deposition of glycogen can further exacerbate myocardial fibrosis and thus cardiac dysfunction. This effect of glycogen deposition provides another potential explanation for why the obese diabetic mice developed heart systolic dysfunction earlier than did the non-obese diabetic mice.

Oxidative stress is the main contributing factor to the tissue damage caused by lipotoxicity 54,60. Under lipotoxicity, tissues take up large amounts of FFAs, leading to the excessive oxidation of FFAs and, as a consequence, a dramatic increase in oxygen consumption and the generation of large amounts of ROS. In addition, excessive FFAs uptake and oxidation result in large increases in intermediate metabolites and upregulated ceramide synthesis, which lead to increased cardiomyocyte apoptosis via the mitochondrial pathway. In addition, excessive FFAs oxidation significantly increases myocardial oxygen consumption and ROS production, thereby inducing oxidative stress and leading to myocardial injury 4,60. The Nrf2 pathway is closely related to the anti-oxidative effect and is activated upon the initiation of oxidative stress 61. In the present study, although oxidative stress was observed in both models, the expression of nuclear Nrf2 and its target genes HO-1 and NQO1 was significantly increased in the hearts of the obese T2DM mice but decreased in the hearts of the non-obese T2DM mice at the time of systolic dysfunction. These results reveal the differences in the factors leading to oxidative stress in the hearts of the obese and non-obese diabetic mice, and the decrease in the expression of antioxidant molecules in the hearts of non-obese diabetic mice may be an important factor that leads to oxidative stress. Moreover, these results may provide guidance for clinical treatment. Enhanced antioxidant ability may have more obvious benefits in the hearts of non-obese T2DM patients than in those of obese T2DM patients. In addition, apoptosis was observed in the hearts of both T2DM models, although the obese T2DM mice exhibited higher levels of apoptosis and caspase-3 activation. We speculate that this result may be related to the more dramatic myocardial lipotoxicity in the obese T2DM mice.

In this study, we systematically compared myocardial injury in obese and non-obese diabetic mice, including changes in the structure, function, energy metabolism, oxidative stress adaptations and related molecular mechanisms. The results showed that obese diabetic mice developed systolic dysfunction earlier than did the non-obese diabetic mice and that the structure, function, energy metabolism, oxidative stress adaptations and related molecular mechanisms were dramatically different between the obese and non-obese diabetic mice (Figure S7). More importantly, some important results from the animal models were clearly validated in human samples. The results from the human samples further demonstrated that the level of lipotoxicity was significant in the hearts of the obese T2DM patients but not in the hearts of the non-obese T2DM patients. Moreover, the obese T2DM patients exhibited more severe cardiac remodeling than did their non-obese counterparts.

In summary, our study confirms that myocardial lipotoxicity and antioxidant dysfunction are key factors in myocardial injury in obese and non-obese T2DM patients, respectively. On the one hand, obese patients with T2DM are more likely to develop myocardial injury than are non-obese patients with T2DM due to persistent and severe myocardial lipotoxicity. On the other hand, severe systolic dysfunction is induced by antioxidant dysfunction in the non-obese T2DM patients. This study is the first systematic comparison of the pathophysiological differences in type 2 diabetic myocardial injury between obese and non-obese models. The findings suggest that myocardial injury in patients with T2DM can be categorized based on metabolic and antioxidant adaptations. More importantly, our study provides a convenient method for the accurate diagnosis of myocardial injury in T2DM patients by distinguishing myocardial injury in obese and non-obese T2DM patients according to BMI or abdominal fat thicker. To date, no relevant research has been presented on the classification of myocardial injury in T2DM patients, which means that the treatment of patients with different types of DCM in T2DM may lack sufficient pertinence. Our research fills this gap and provides insights into the differences in DCM between obese and non-obese T2DM patients, and these findings lay the foundation for developing more targets and precise treatments for DCM. On the one hand, reducing lipotoxicity in the treatment of myocardial injury in obese T2DM patients may lead to better outcomes than can be expected for non-obese T2DM patients with systolic dysfunction or heart failure. On the other hand, increasing the antioxidant capacity is important in the treatments for both obese and non-obese T2DM patients with myocardial injury; however, this strategy will likely have a more powerful amelioration benefit in the non-obese T2DM patients (Figure S8). Our research shows that more accurate disease characterization may not be limited to the diabetes itself. For the same type of diabetes, complications in the same organ may have many significant differences which that are important for appropriate phenotyping. Understanding these differences enables medical professionals to help patients receive more accurate diagnoses and therapies.

Despite the clear and definite implications of our research, certain shortcomings should be noted. First, whereas diabetes and obesity in humans are predominantly polygenic and multifactorial, the mouse model used in this study presents with a monogenic form of diabetes and obesity; thus, it is fundamental to use more mouse models (e.g., the NZO polygenic diabetic and obese mice) to correlate the human and mouse observations. Second, since cardiac samples were not readily available, our research involved only samples from humans without systolic dysfunction or heart failure; therefore, our conclusions need to be validated clinically - or at least in samples of human hearts from people with systolic dysfunction or heart failure. Finally, although we demonstrated that different energy metabolism and oxidative stress adaptations are the key factors leading to the differences in DCM between the obese and non-obese mice with T2DM, we did not evaluate the appropriateness of any intervention; therefore, further research is warranted to evaluate the therapeutic effects of different treatment strategies on DCM patients with different BMIs.

## Supplementary Material

Supplementary figures and tables.
