# Supplementary material for: Distinct cardiac energy metabolism and oxidative stress adaptations between obese and non-obese type 2 diabetes mellitus
Source: Theranostics. 2020 Feb 3;10(6):2675–95. doi: 10.7150/thno.40735 (PMC7052888; doi:10.7150/thno.40735)
Supplement: Supplementary file 1 — Supplementary figures and tables. [file thnov10p2675s1.pdf]

Supplementary Material

Supplementary Figure Legends

Figure S1. Identification of T2DM mice model with obesity or non-obesity. (A) Roadmap of mouse model building (T2DM with obesity). (B) Growth curve of T2DM mice with obesity. (n = 10-30) (C) Blood glucose of T2DM mice with obesity. (n = 10-30) (D) Serum insulin content of T2DM mice with obesity. (n = 6) (E) HOMO-IR of T2DM mice with obesity. (n = 6) (F) ITT of T2DM mice with obesity in the 4th week of the disease course. (n = 6) (G) Roadmap of mouse model building (T2DM with non-obesity). (H) Growth curve of T2DM mice with non-obesity. (n = 9-50) (I) Blood glucose of T2DM mice with non-obesity. (n = 9-38) (J) Serum insulin content of T2DM mice with non-obesity. (n = 6) (K) HOMO-IR of T2DM mice with non-obesity. (n = 6) (L) GTT of T2DM mice with non-obesity in the 4th week of the disease course. (n = 6) (M) ITT of T2DM mice with non-obesity in the 24th week of the disease course. (n = 6) (N) HE staining of islet tissues in the 24th and 36th week of the disease course (Scale bar = 50 μm). Data are expressed as mean ± SD. (*, $p < 0.05$; **, $p < 0.01$; ***, $p < 0.001$.).

Figure S2. The heart rate and cardiac diastolic function of obese and non-obese T2DM mice. (A) Heart rate of obese mice in the 24th week of the disease course. (B) Mitral valve rheography of obese mice in the 24th week of the disease course. (C) E/A ratio of obese mice in the 24th week of the disease course. (D) Aortic ejection time of obese mice in the 24th week of the disease course. (E) Isovolumetric contraction time of obese mice in the 24th week of the disease course. (F) Isovolumic relaxation time of obese mice in the 24th week of the disease course. (G) Heart rate of non-obese mice in the 36th week of the disease course before and after anesthesia. (H) Mitral valve rheography of non-obese mice in the 24th and 36th week of the disease course. (I) E/A ratio of non-obese mice in the 24th and 36th week of the disease course. (J) Aortic ejection time of non-obese mice in the 24th and 36th week of the disease course. (K) Isovolumetric contraction time of non-obese mice in the 24th and 36th week of the disease course. (L) Isovolumic relaxation time of non-obese mice in the 24th and 36th week of the disease course. Data are expressed as mean ± SD. (*, $p < 0.05$; **, $p < 0.01$; ***, $p < 0.001$. n = 6-10 for each group).

Figure S3. Serum lipids of obese and non-obese T2DM mice. (A) Serum FFAs content of obese T2DM mice. (B) Serum TG content of obese T2DM mice. (C) Serum TC content of obese T2DM mice. (D) Serum LDL content of obese T2DM mice. (E) Serum HDL content of obese T2DM mice. (F) Serum FFAs content of non-obese T2DM mice. (G) Serum TG content of non-obese T2DM mice. (H) Serum TC content of non-obese T2DM mice. (I) Serum LDL content of non-obese T2DM mice. (J) Serum HDL content of non-obese T2DM mice. Data are expressed as mean ± SD. (*, $p < 0.05$; **, $p < 0.01$; ***, $p < 0.001$. n = 6 for each group).

Figure S4. Western blot results of proteins related to insulin resistance and glycogen synthesis in heart tissues from T2DM mice. (A) Western blot images of proteins related to Akt pathway in hearts tissues from obese T2DM mice. (B) Relative quantification based on the panel A. (C) Western blot images of proteins related to Akt pathway in hearts tissues from non-obese T2DM mice. (D) Relative quantification based on the panel C. Data are expressed as mean ± SD. (*, $p < 0.05$; **, $p < 0.01$; ***, $p < 0.001$. n = 3 for each group)

Figure S5. Western blot results of proteins related to oxidative stress and apoptosis in heart tissues from obese T2DM mice. (A) Western blot images of proteins related to oxidative stress and apoptosis in heart tissues from obese T2DM mice. (B) Relative quantification based on the results of panel A. Data are expressed as mean ± SD. (*，$p < 0.05$; **，$p < 0.01$; ***，$p < 0.001$. n = 3 for each group)

Figure S6. Western blot results of proteins related to oxidative stress and apoptosis in heart tissues from non-obese T2DM mice. (A) Western blot images of proteins related to oxidative stress and apoptosis in heart tissues from non-obese T2DM mice. (B) Relative quantification based on the results of panel A. Data are expressed as mean ± SD. (*，$p < 0.05$; **，$p < 0.01$; ***，$p < 0.001$. n = 3 for each group)

Figure S7. The summary picture of the results in T2DM mice. The summary picture showing the differences of cardiac function and molecular mechanism in hearts of T2DM mice with obesity or non-obesity through the disease course.

Figure S8. Competency in medical knowledge and translational outlook. Obese T2DM exhibits more severe and sustained lipotoxicity compared to non-obese T2DM. Non-obese T2DM exhibits a significant decrease in antioxidant capacity in the event of systolic dysfunction compared to obese T2DM. Obese T2DM is more prone to cardiac systolic dysfunction due to different cardiac energy metabolism and oxidative stress adaptations in T2DM with obese and non-obese. Different BMI can be used as a basis for judging energy intervention or antioxidant therapy.

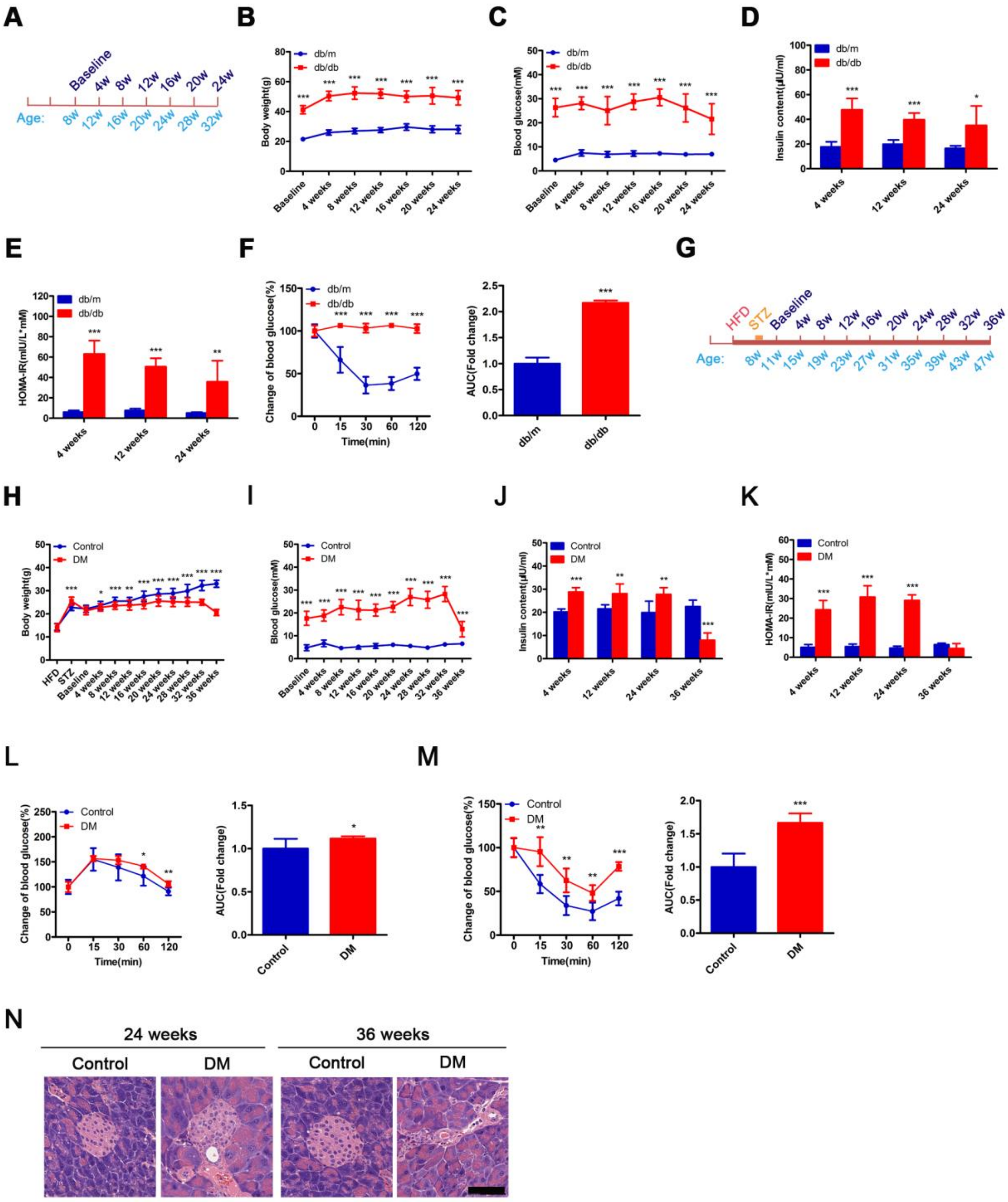

A
Baseline
4w
8w
12w
16w
20w
24w
Age:
8w
12w
16w
20w
24w
28w
32w
B
db/m
db/db
Body weight(g)
Baseline
4 weeks
8 weeks
12 weeks
16 weeks
20 weeks
24 weeks
C
db/m
db/db
Blood glucose(mM)
D
db/m
db/db
Insulin content(μU/ml)
E
db/m
db/db
HOMA-IR(mIU/L*mM)
F
db/m
db/db
Change of blood glucose(%)
Time(min)
AUC(Fold change)
G
HFD
STZ
Baseline
Age:
H
Control
DM
Body weight(g)
I
Control
DM
Blood glucose(mM)
J
Control
DM
Insulin content(μU/ml)
K
Control
DM
HOMA-IR(mIU/L*mM)
L
Control
DM
Change of blood glucose(%)
Time(min)
AUC(Fold change)
M
Control
DM
Change of blood glucose(%)
Time(min)
AUC(Fold change)
N
24 weeks
36 weeks
Control
DM
Control
DM

Figure S1

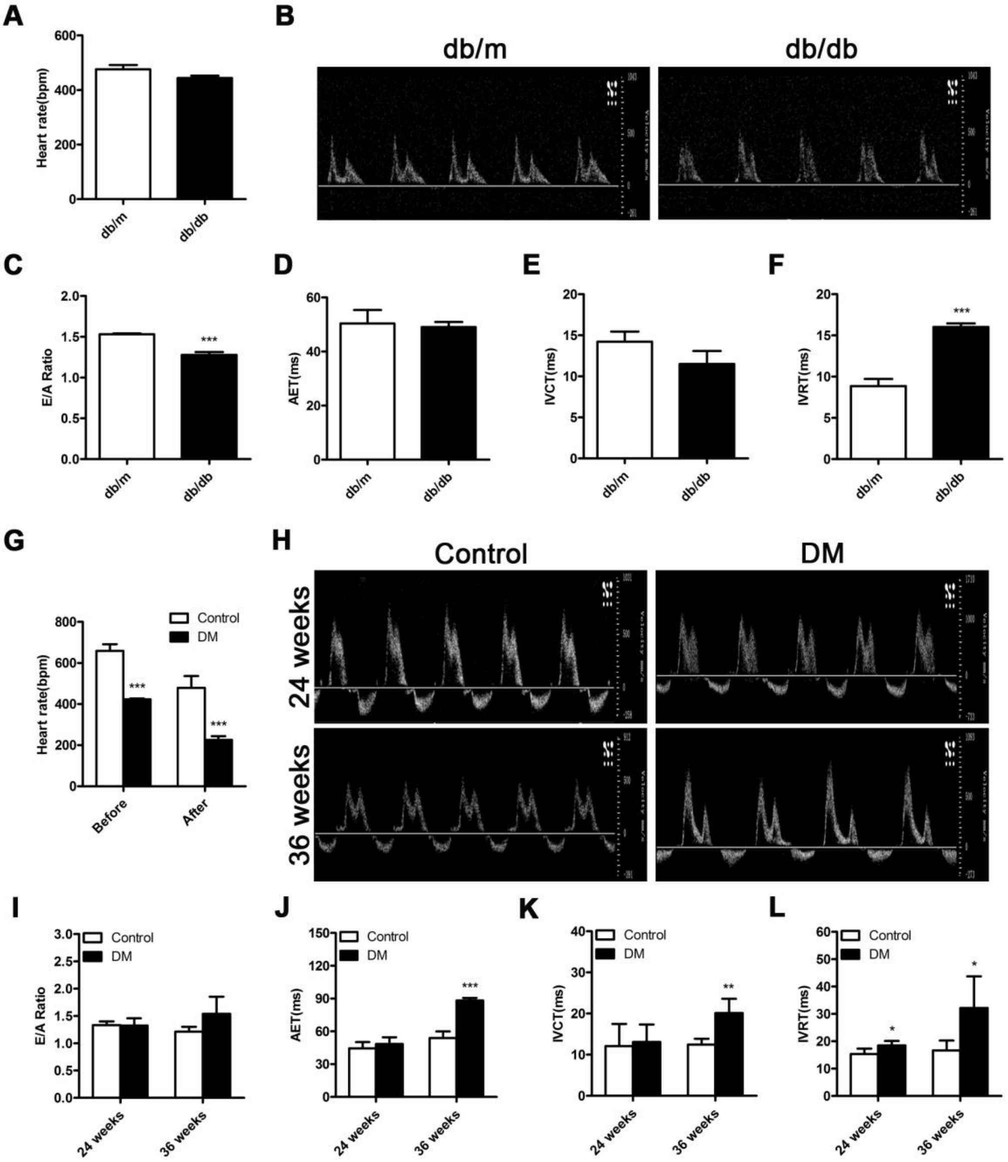
A
B
db/m
db/db
Heart rate(bpm)
C
D
E
F
E/A Ratio
AET(ms)
IVCT(ms)
IVRT(ms)
***
G
H
Control
DM
24 weeks
36 weeks
Before
After
I
J
K
L
**
*

Figure S2

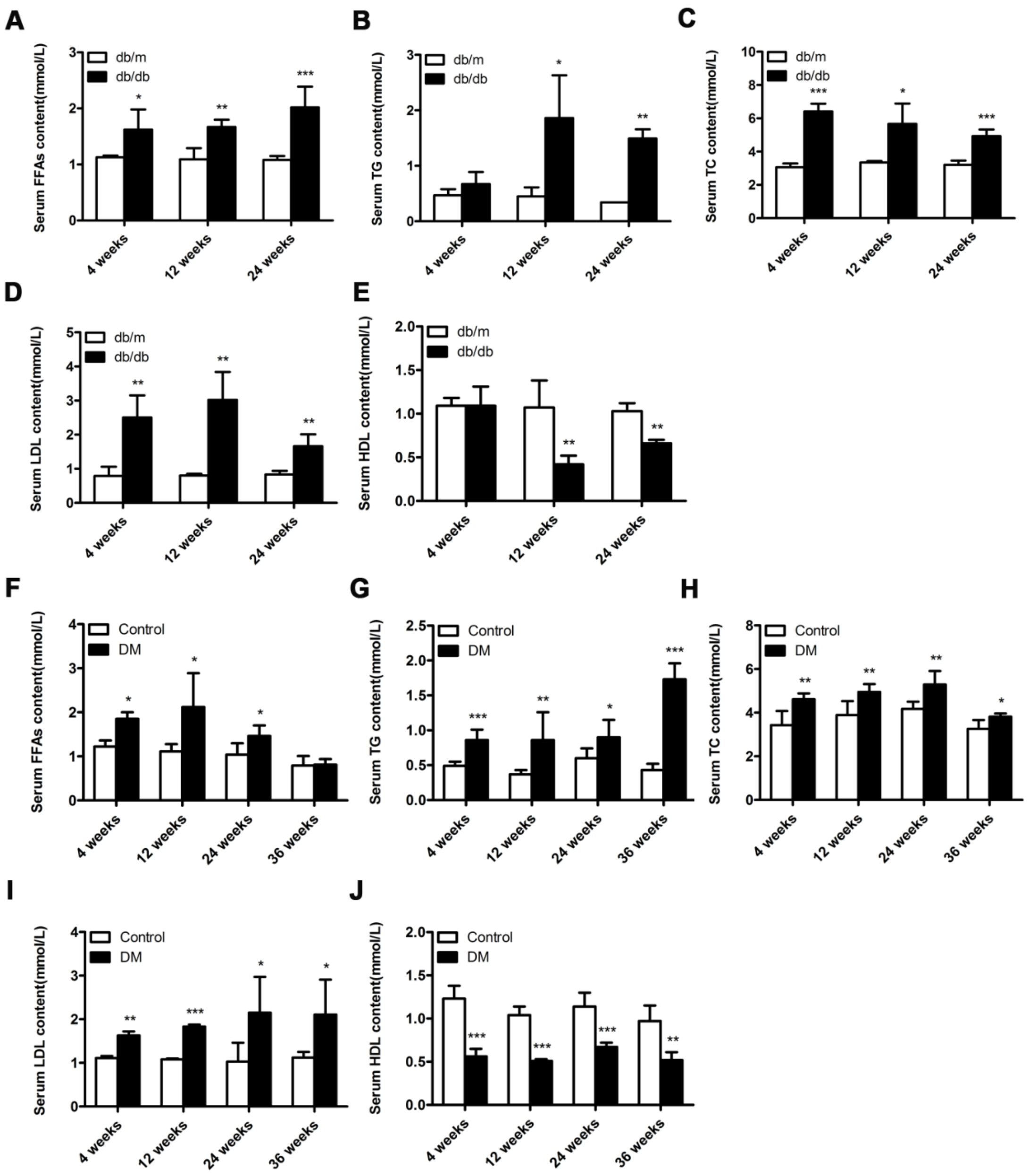
A
Serum FFAs content(mmol/L)
db/m
db/db
4 weeks
12 weeks
24 weeks
B
Serum TG content(mmol/L)
C
Serum TC content(mmol/L)
D
Serum LDL content(mmol/L)
E
Serum HDL content(mmol/L)
F
Control
DM
36 weeks
G
H
I
J

Figure S3

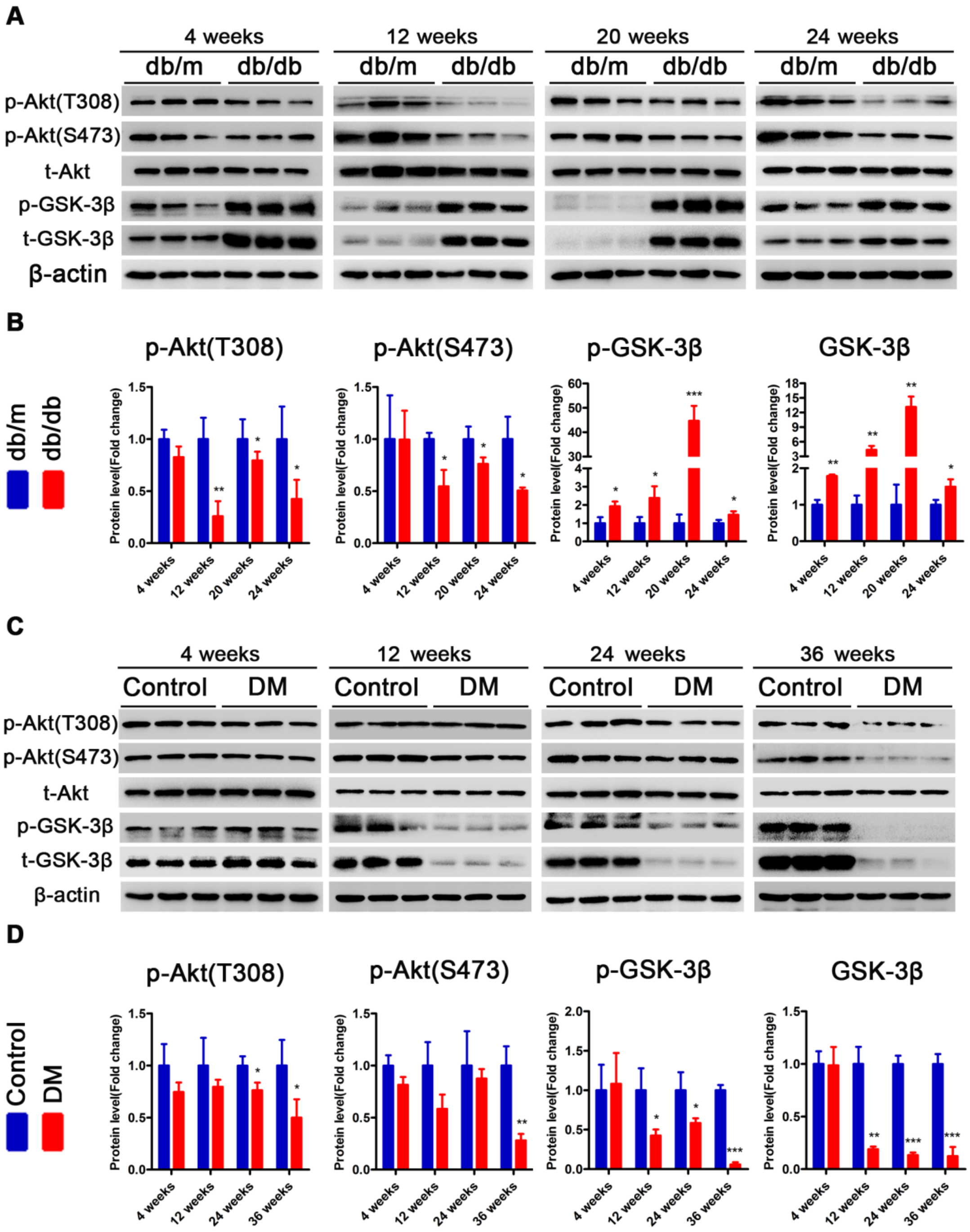
A
4 weeks
12 weeks
20 weeks
24 weeks
db/m
db/db
p-Akt(T308)
p-Akt(S473)
t-Akt
p-GSK-3β
t-GSK-3β
β-actin
B
p-Akt(T308)
p-Akt(S473)
p-GSK-3β
GSK-3β
db/m
db/db
Protein level(Fold change)
C
4 weeks
12 weeks
24 weeks
36 weeks
Control
DM
p-Akt(T308)
p-Akt(S473)
t-Akt
p-GSK-3β
t-GSK-3β
β-actin
D
p-Akt(T308)
p-Akt(S473)
p-GSK-3β
GSK-3β
Control
DM
Protein level(Fold change)

Figure S4

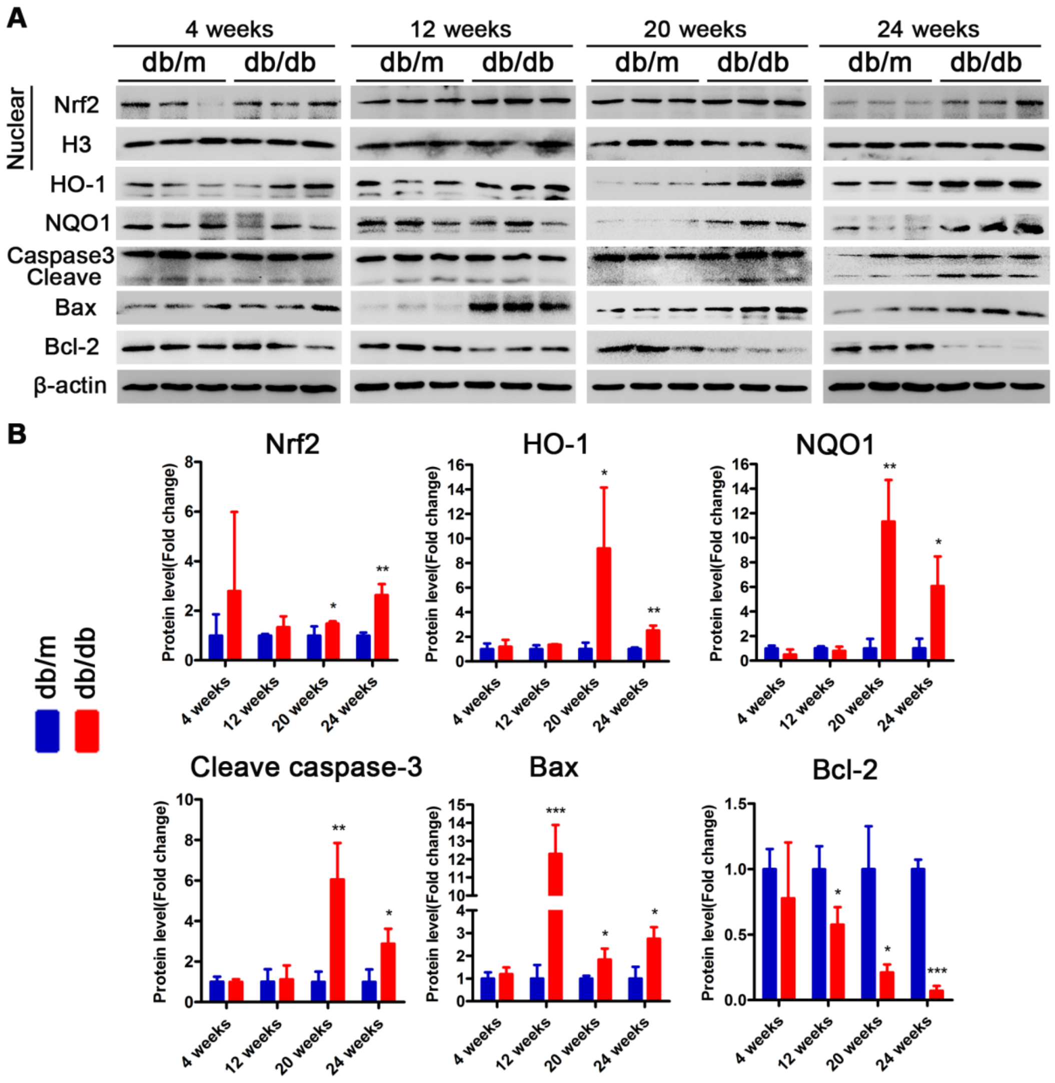
A
4 weeks
12 weeks
20 weeks
24 weeks
db/m
db/db
Nuclear
Nrf2
H3
HO-1
NQO1
Caspase3
Cleave
Bax
Bcl-2
β-actin
B
Nrf2
HO-1
NQO1
Cleave caspase-3
Bax
Bcl-2
Protein level(Fold change)
4 weeks
12 weeks
20 weeks
24 weeks
db/m
db/db

Figure S5

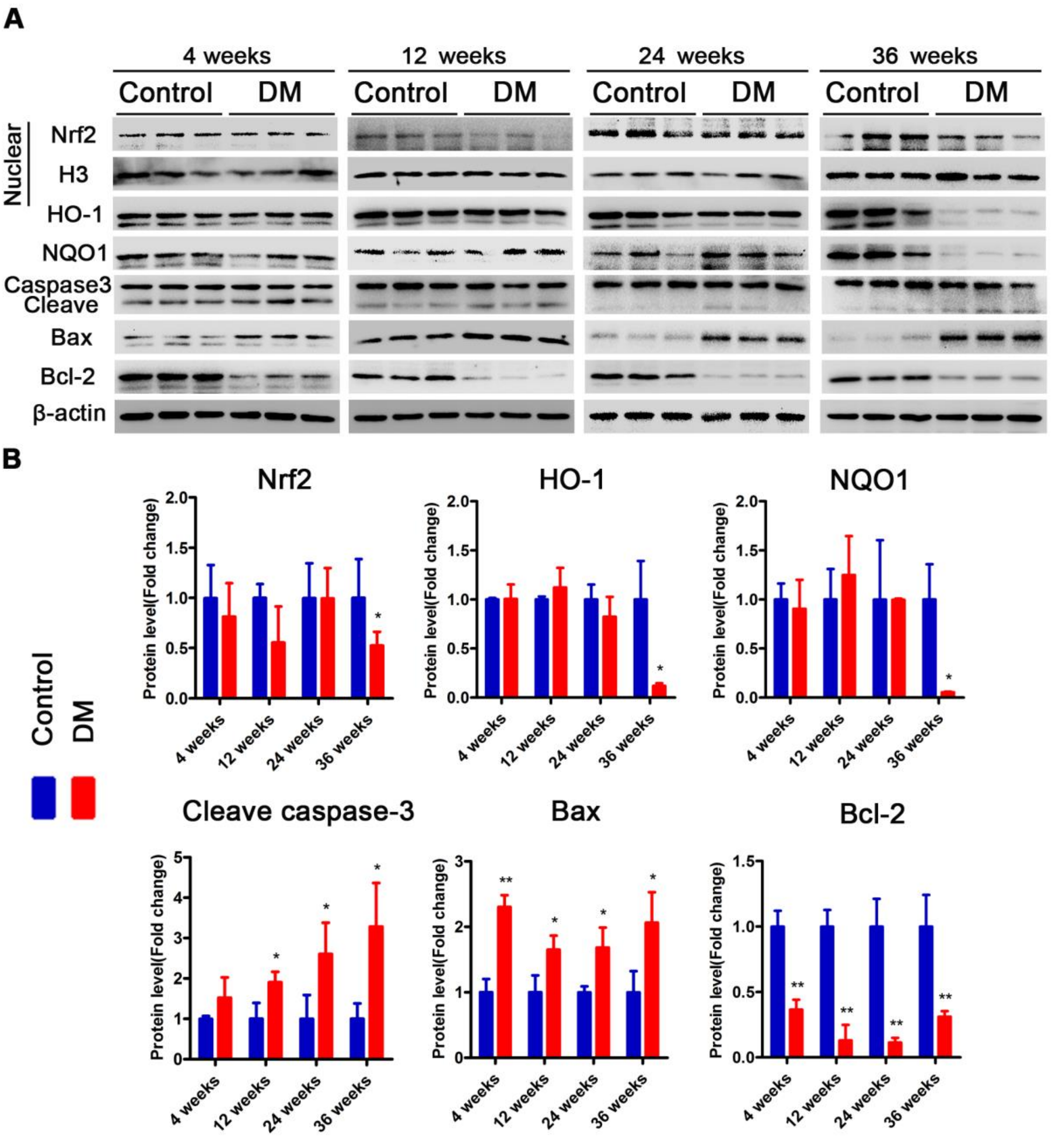
A
4 weeks
12 weeks
24 weeks
36 weeks
Control
DM
Nuclear
Nrf2
H3
HO-1
NQO1
Caspase3
Cleave
Bax
Bcl-2
β-actin
B
Nrf2
HO-1
NQO1
Cleave caspase-3
Bax
Bcl-2
Protein level(Fold change)
4 weeks
12 weeks
24 weeks
36 weeks
Control
DM

Figure S6

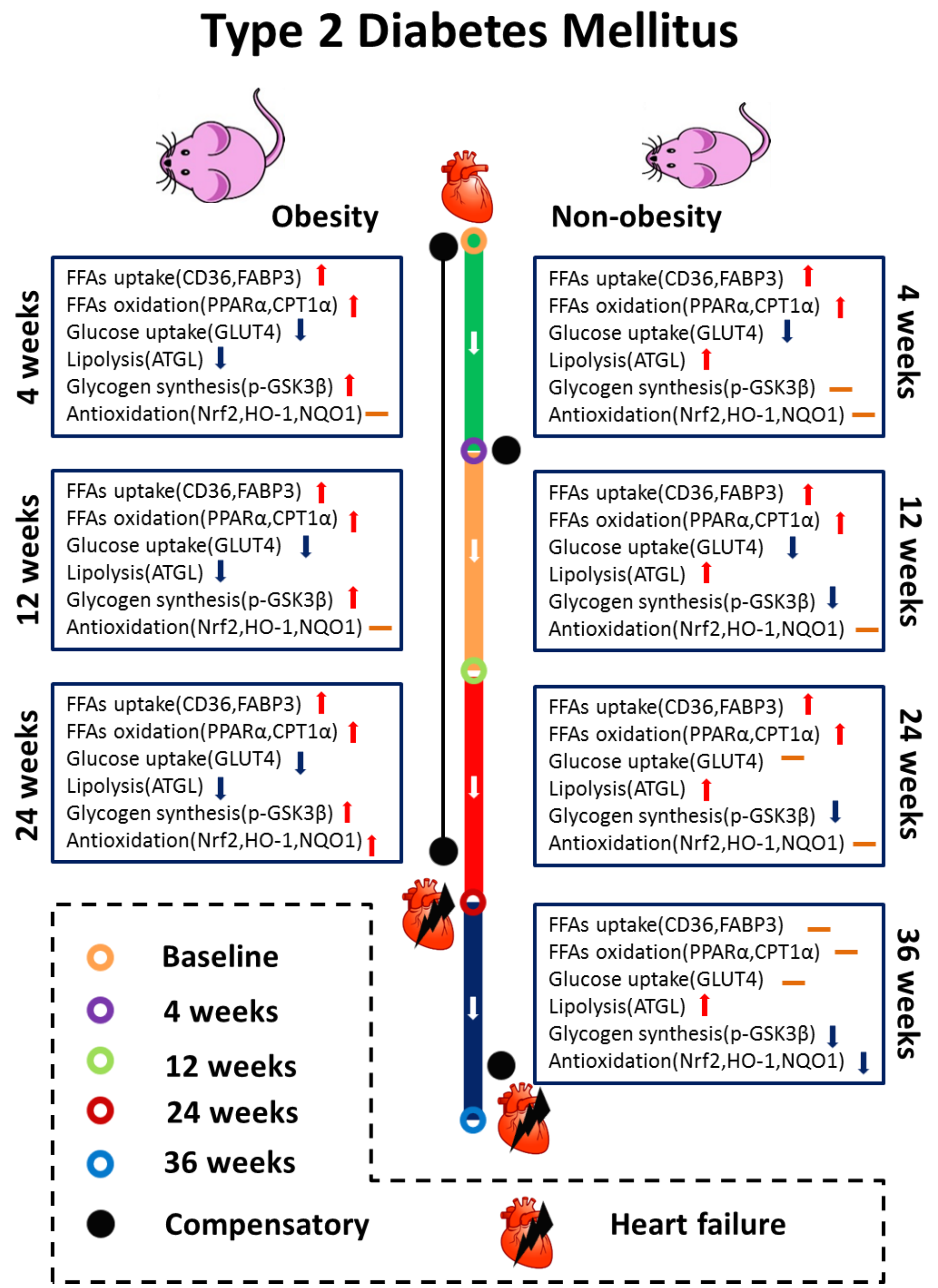
Type 2 Diabetes Mellitus
Obesity
Non-obesity
4 weeks
FFAs uptake(CD36,FABP3)
FFAs oxidation(PPARα,CPT1α)
Glucose uptake(GLUT4)
Lipolysis(ATGL)
Glycogen synthesis(p-GSK3β)
Antioxidation(Nrf2,HO-1,NQO1)
12 weeks
24 weeks
36 weeks
Baseline
4 weeks
12 weeks
24 weeks
36 weeks
Compensatory
Heart failure

Figure S7

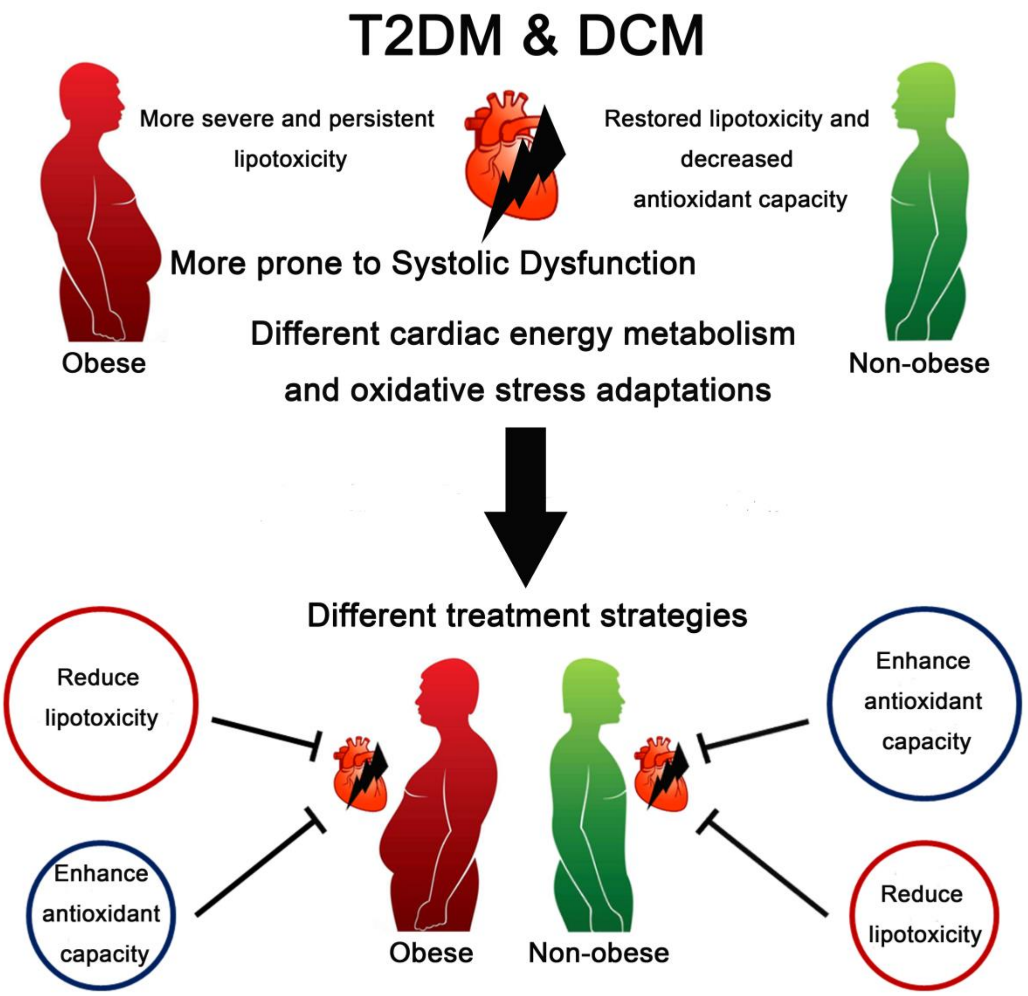
T2DM & DCM
More severe and persistent lipotoxicity
Restored lipotoxicity and decreased antioxidant capacity
More prone to Systolic Dysfunction
Obese
Non-obese
Different cardiac energy metabolism and oxidative stress adaptations
Different treatment strategies
Reduce lipotoxicity
Enhance antioxidant capacity
Enhance antioxidant capacity
Reduce lipotoxicity
Obese
Non-obese

Figure S8

Table S1. The information of antibodies.

Antibodies	Manufacture and item NO.	Application
CD31	Abcam(ab28364)	Immunofluorescence
PPARα	Abcam(ab8934)	Western blotting
CD36	Sigma(b104094)	Western blotting
FABP3	Cell signaling technology(14780S)	Western blotting
FATP4	Sigma(SAB2102194)	Western blotting
CPT1α	Abcam(ab128568)	Western blotting
ATGL	Cell signaling technology(2439S)	Western blotting, IHC
GLUT4	Cell signaling technology(2213S)	Western blotting
p-Akt(T308)	Cell signaling technology (13038S)	Western blotting
p-Akt(S73)	Cell signaling technology (4060S)	Western blotting
Akt	Cell signaling technology (4691S)	Western blotting
p-GSK-3β	Cell signaling technology (5558S)	Western blotting, IHC
GSK-3β	Cell signaling technology (12456S)	Western blotting
Nrf2	Cell signaling technology (12721S)	Western blotting
HO-1	Cell signaling technology (70081S)	Western blotting, IHC
NQO1	Abcam (ab28947)	Western blotting
Histone H3	Cell signaling technology (4499S)	Western blotting
(Cleave)Caspase3	Cell signaling technology (9915T)	Western blotting
Bax	Beyotime (AB026)	Western blotting
Bcl-2	Beyotime (AB112)	Western blotting
β-actin	Cell signaling technology (3700S)	Western blotting
FABP3	Proteintech (10676-1-AP)	IHC
CPT-1α	Proteintech (15184-1-AP)	IHC
NQO1	Proteintech (11451-1-AP)	IHC

Table S2. Primers for qPCR.

Genes	Primers
Mouse ANP	F: 5′-GCTTCCAGGCCATATTGGAGCA-3′
	R: 5′-TCTCTCAGAGGTGGGTTGACCT-3′
Mouse BNP	F: 5′-ATGGATCTCCTGAAGGTGCTGT-3′
	R: 5′-GCAGCTTGAGATATGTGTCACC-3′
Mouse Acadvl	F: 5′-ATCAGGTGTTCCCATACCCA-3′
	R: 5′-TCCTTGAGTCCCTGCAAAGT-3′
Mouse Acsl1	F: 5′-AACGAGGCAAGAAGTGTGGG-3′
	R: 5′-TGGTGAGTGATCATTGCTCC-3′
Mouse Acadm	F: 5′-AGGATGACGGAGCAGCCAAT-3′
	R: 5′-ATCTGGGTTAGAACGTGCCA-3′
Mouse Cpt1β	F: 5′-CGTTCACGCCATGATCATGT-3′
	R: 5′-AGAGCCAGACCTTGAAGAAG-3′
Mouse Acaa2	F: 5′-TCAACAGGCTCTGTGGCTCT-3′
	R: 5′-TGCCCACAAAGTATCTTCC-3′
Mouse Fabp3	F: 5′- CTGGACGGAGGCAAACTCAT-3′
	R: 5′- AGGGGAAAACCATGAGGCAG-3′
Mouse CD36	F: 5′- GATGAGCATAGGACATACTTAGATGTG-3′
	R: 5′- CACCACTCCAATCCCAAGTAAG-3′
Mouse GAPDH	F: 5’- TGCGACTTCAACAGCAACTC-3’
	R: 5’- GCCTCTCTTGCTCAGTGTCC-3’

Table S3. The information of donors.

No.	Type	Gender	Age (Years)	Abdominal fat thickness(cm)	Heart weight (g)
1	T2DM with obesity	Male	43	3.5	380
2		Male	55	3.4	500
3		Female	68	4	350
4		Male	49	11	700
5		Female	45	3	400
6		Female	63	3	450
7	Healthy population (Control group for T2DM with obesity)	Male	41	1.5	350
8		Male	55	1.5	350
9		Female	68	2.1	300
10		Male	51	1.2	420
11		Female	44	2.7	300
12		Female	64	1.8	350
13	T2DM with non-obesity	Male	64	1.5	500
14		Male	50	1.2	600
15		Female	68	1.4	400
16		Female	66	1	350
17		Male	41	1	380
18		Male	72	2	520
19		Male	49	1.3	300
20		Female	70	2.4	440
21	Healthy population (Control group for T2DM with non-obesity)	Male	64	2	400
**10**		Male	51	1.2	420
**9**		Female	68	2.1	300
**12**		Female	64	1.8	350
22		Male	41	0.8	380
23		Male	71	1	300
24		Male	49	2	450
25		Female	68	1.6	300
